# Beyond pros and cons – developing a patient decision aid to cultivate dialog to build relationships: insights from a qualitative study and decision aid development

**DOI:** 10.1186/s12911-019-0898-5

**Published:** 2019-09-18

**Authors:** Catherine H. Yu, Calvin Ke, Aleksandra Jovicic, Susan Hall, Sharon E. Straus, on behalf of the IP-SDM Team;, Paul Cantarutti, Karen Chu, Paul Frydrych, Noah Ivers, David Kaplan, Fok-Han Leung, John Maxted, Jeremy Rezmovitz, Sumeet Sodhi, Deanna Telner, Joanna Sale, Li Ka Shing, Dawn Stacey, Sharon Straus

**Affiliations:** 1 Li Ka Shing Knowledge Institute of St. Michael’s Hospital, 30 Bond Street, Toronto, ON M5B 1W8 Canada; 2 Department of Medicine, University of Toronto, 190 Elizabeth Street, Toronto, ON M5G 2C4 Canada; 3 Dalla Lana School of Public Health, University of Toronto, 155 College St, Toronto, ON M5T 3M7 Canada; 4 Flexxis R&D Consulting, Toronto, Canada; 5 Department of Health Policy Management and Evaluation, University of Toronto, Toronto, ON Canada

**Keywords:** Shared decision-making, Priority-setting, Patient decision aid, Interprofessional care, Diabetes mellitus, Patient education, Medical informatics, Toolkit development, User-centred design, Qualitative methods

## Abstract

**Background:**

An individualized approach using shared decision-making (SDM) and goal setting is a person-centred strategy that may facilitate prioritization of treatment options. SDM has not been adopted extensively in clinical practice. An interprofessional approach to SDM with tools to facilitate patient participation may overcome barriers to SDM use. The aim was to explore decision-making experiences of health professionals and people with diabetes (PwD), then develop an intervention to facilitate interprofessional shared decision-making (IP-SDM) and goal-setting.

**Methods:**

This was a multi-phased study. *1) Feasibility*: Using a descriptive qualitative study, individual interviews with primary care physicians, nurses, dietitians, pharmacists, and PwD were conducted. The interviews explored their experiences with SDM and priority-setting, including facilitators and barriers, relevance of a decision aid for priority-setting, and integration of SDM and a decision aid into practice. *2) Development*: An evidence-based SDM toolkit was developed, consisting of an online decision aid, *MyDiabetesPlan*, and implementation tools. *MyDiabetesPlan* was reviewed by content experts for accuracy and comprehensiveness. Usability assessment was done with *3) heuristic evaluation* and *4) user testing*, followed by *5) refinement*.

**Results:**

Seven PwD and 10 clinicians participated in the interviews. From interviews with PwD, we identified that: (1) approaches to decision-making were diverse and dynamic; (2) a trusting relationship with the clinician and dialog were critical precursors to SDM; and, (3) goal-setting was a dynamic process. From clinicians, we found: (1) complementary (holistic and disease specific) approaches to the complex patient were used; (2) patient-provider agendas for goal-setting were often conflicting; (3) a flexible approach to decision-making was needed; and, (4) conflict could be resolved through SDM. Following usability assessment, we redesigned *MyDiabetesPlan* to consist of data collection and recommendation stages. Findings were used to finalize a multi-component toolkit and implementation strategy, consisting of *MyDiabetesPlan*, instructional card and videos, and orientation meetings with participating patients and clinicians.

**Conclusions:**

A decision aid can provide information, facilitate clinician-patient dialog and strengthen the therapeutic relationship. Implementation of the decision aid can fit into a model of team care that respects and exemplifies professional identity, and can facilitate intra-team communication.

**Trial registration:**

Clinicaltrials.gov Identifier: NCT02379078. Date of Registration: 11 February 2015.

**Electronic supplementary material:**

The online version of this article (10.1186/s12911-019-0898-5) contains supplementary material, which is available to authorized users.

## Background

Patients with complex chronic diseases are presented with competing disease priorities; competing patient-physician priorities further complicate care. Effective shared decision making (SDM) tools have been adapted for use in chronic care including diabetes [1, 2] and may enable prioritization of treatment options. With SDM, patients and clinicians establish an ongoing partnership, exchange information, deliberate on options, decide upon the priority for taking action, and then act on the decision [3]. A systematic review of randomized controlled trials of SDM identified 11 studies [4], five of which examined physical and psychological wellbeing. Two of these studies reported positive outcomes; these two studies examined long-term decisions in the setting of chronic disease, suggesting that SDM may play an important role in complex diabetes care. A more recent systematic review of SDM in older people identified 22 studies, two in patients with diabetes, and found that SDM increased knowledge, increased risk perception, reduced decisional conflict and enhanced participation in SDM [5].

Despite this evidence supporting the role of SDM in complex diabetes care, the integration of SDM into clinical practice is limited. Specific to diabetes, patients have reported that patient/clinician power imbalance, health literacy, and denial of the condition were barriers to SDM. Provision of medical knowledge, validation of patient experiences, strong interpersonal skills, and clinician availability were facilitators of SDM [6].

An interprofessional (IP) team approach may overcome these barriers to SDM. Interprofessional care, where professionals from different disciplines collaborate to provide an integrated approach to patient care [7] is particularly appropriate for diabetes care. Participation by more than one profession, expanding roles, and adding new team members in diabetes care has been demonstrated to improve clinical outcomes [8–10] and may increase uptake of SDM [11].

Furthermore, SDM can be facilitated by the use of patient decision aids (PtDAs) [12–14]. For example, an observational study by Corser et al. showed that using a PtDA to support goal-setting in diabetes care increased patient knowledge (*P* = 0.001) as well as the number of documented diabetes goals (pre: 0.67 goals; post: 1.09 goals; *P* < 0.001), though there was no change in glycemic control, weight, or diabetes empowerment score [15]. However, this study did not examine an interprofessional approach nor specifically integrate principles of shared decision-making.

Building on this study, we hypothesized that a multi-component SDM toolkit (patient-directed, clinician-directed, and point-of-care tools) that individualized care priorities and incorporated an IP approach to SDM would help to prioritize complex guideline recommendations for patients with type 1 or type 2 diabetes and other comorbidities. The aim was to explore decision-making experiences of health professionals and patients with diabetes and co-morbidities, then develop an intervention employing user-centred design to facilitate IP-SDM and goal-setting.

## Methods

This study was part of a larger study focused on developing and evaluating an SDM toolkit for goal setting in patients with diabetes and other comorbidities; the development and evaluation protocol is described in detail elsewhere [16]. Briefly, we used the Knowledge to Action (KTA) Framework [17] to design and evaluate a multi-component SDM toolkit. This toolkit consisted of a PtDA (*MyDiabetesPlan*) and its accompanying implementation tools (such as how-to videos, and enabler cards with step-by-step instructions). It was developed to help prioritize guideline-based disease management in patients with multiple comorbidities, defined as those with type 1 or type 2 diabetes and two additional chronic conditions. At the outset, we engaged a multi-disciplinary research team, including people with diabetes, family physicians, nurses, and content experts with expertise in decision aid development and evaluation, qualitative and quantitative methodology. We also used the Medical Research Council framework for the development and evaluation of complex interventions [18]; this combined model was particularly relevant to our intervention since it relies on multiple interacting components, behaviours and groups [19].

### Study overview

Development and evaluation of the SDM toolkit consisted of 4 phases: [1] feasibility testing; [2] toolkit development; [3] heuristic evaluation; and [4] usability testing. Throughout the development process, the toolkit was refined iteratively based on findings of each phase. We used the Consolidated Checklist for Reporting Qualitative Research (COREQ) checklist [20] (Additional file 1: Table S1) to ensure reporting rigour and the International Patient Decision Aids Standards (IPDAS) checklist [21] to ensure decision aid development rigour (Additional file 1: IPDAS checklist).

### Phase 1: feasibility testing

Individual semi-structured interviews [22] with clinicians and patients were used to explore their experiences with priority-setting and SDM including their facilitators and barriers, the utility of a PtDA and toolkit for priority-setting, what content to include in the PtDA and how to integrate these into practice. Participants then worked through a prototype of the PtDA while the interviewer provided scripted responses, acting as either the “patient” or “clinician”.

#### Participants

Using purposive sampling [23], family physicians, as well as nurses, dietitians, and/or pharmacists (either certified diabetes educators or not) with varied profiles (age, gender) were recruited through family health teams in the academic and community settings in the Greater Toronto Area and surrounding regions.

Patients with type 1 or type 2 diabetes and two other comorbidities including: heart disease (e.g. ischemic, valvular, congestive, arrhythmic, congenital disease), stroke, hypertension, cancer (excluding non-melanoma skin cancer), chronic lung disease, arthritis, inflammatory bowel disorders, and urinary incontinence and with varied socio-demographic profiles (gender, educational attainment) in these family health teams were identified through their clinicians or chart review. Exclusion criteria were: inability to speak English or provide consent, pregnancy or considering pregnancy.

#### Data collection

Individual interviews with patients and clinicians were conducted using semi-structured interview guides developed by team members with expertise in SDM and qualitative methods. The interview guides were based on pre-existing literature regarding approaches to multiple comorbidities, barriers and facilitators to SDM and goal-setting [6, 14], and the Theoretical Domains Framework [24, 25]. We also explored barriers and facilitators to PtDA adoption and preferences for format and content (Additional file 1: Interview Guide).

Participants then used a web-based decision-aid prototype created by the principal investigator with content from evidence-based guidelines [26] and International Patient Decision Aid Standards checklist [21]. During this component of the session, the interviewer played the role of either the clinician or patient using scripted responses while the participant used the prototype (e.g. if the participant was a clinician, then the interviewer responded as if she was a person with diabetes, and vice versa). We chose to conduct role play with a prototype to mimic actual clinical use in order to identify facilitators and barriers that may arise with real use, as well as feedback regarding format and content. Face validity of this guide was assessed with team members (patient, nurse, family physician, endocrinologist) and refined as needed; in addition, it was tested in “practice” interviews on other team members. A trained interviewer conducted each interview at the clinician’s practice (when interviewing clinicians), or at our research site (when interviewing patients). All interviews were audiotaped then transcribed verbatim and annotated with field notes.

#### Analysis

Data analysis was conducted in conjunction with data collection, resulting in iterative refinement of the interview guide. Inductive thematic analytic techniques were used [27–29]. All transcripts were coded for emergent themes [22], reviewed independently by at least two team members and consensus on coding was reached through discussion during. Regular meetings and documented with memos. NVivo software (v.10) was used to organize and store the data.

### Phase 2: *MyDiabetesPlan* and implementation strategy development

#### Overview

Building on facilitators of SDM adoption [14], a goal-setting intervention [15] and the findings of Phase 1, *MyDiabetesPlan* was developed, using the IP-SDM framework [30] following the International Patient Decision Aids Standards criteria [21]. Specifically, *MyDiabetesPlan* elicits the patient’s general care priorities, identifies his/her diabetes-specific goals and outcomes, outlines diabetes-specific therapies, and details population-specific benefits and risks to therapies. Evidence-based recommendations and patient values uncovered in Phase 1 were used to inform its content. The initial *MyDiabetesPlan* was in English and targeted a Grade 8 literacy level [21]. Although we started with a paper-based prototype, due to the number of required inputs and potential management options, as well as complex weighting algorithms to arrive at the tailored management option based on user input, we elected to use a web-based format for our decision aid. Use of *MyDiabetesPlan* within the care team was dependent on the usual roles, responsibilities and processes of care, and the needs of the patient. For example, if the usual process of care was that the patient first saw the clinic nurse followed by the family physician, then this was adapted for the study.

#### Decision aid prioritization methods

The decision aid framework guided patients through the process of selecting goals (for example, avoiding stroke) and options for achieving those goals (for example, taking preventive medications on time). This framework allowed patients to select goals from an ordered list ranked based on clinical and patient-important factors (for example, wanting to preserve motor ability in order to continue participating in outdoor activities). Each of these factors was assigned a relative weighting based on importance, and goals were listed in descending order (with the highest total weighting appearing first). We only included clinical factors evaluated in risk-prediction algorithms based on large population studies [31–38], and each of these factors was assigned a weighting based on the relative importance to complication prevention. The utility of relative weightings in determining a prioritized goal list was evaluated by content experts and further refined based on the iterative development process.

Options were prioritized based on a similar weighting method, and non-applicable options were removed (e.g. losing weight was eliminated for patients with a normal BMI). The goal (selected by the patient in the previous stage) was used to determine weighting for each of the options, with points allotted to those options, which were included as risk factors identified in the previously mentioned risk-prediction algorithms [31–38]. Weightings for these options were adjusted based on the iterative development process.

The PtDA was reviewed by expert clinicians (family physicians, endocrinologists, geriatricians, nurses, dietitians, pharmacists) and patients not involved in its development. Specifically, each completed a report assessing the accuracy, comprehensiveness, balance of perspective, and ease of understanding of *MyDiabetesPlan*. Data from these reports were analyzed, and any discordant responses between PtDA reviewers were discussed and resolved by the research team. Based on this feedback, revisions to *MyDiabetesPlan* were made.

### Phase 3: heuristic evaluation

This and the next phase of the study involved usability evaluation of the tool. The United States Food and Drug Administration recommends incorporating “usability engineering processes during the development of medical devices, focusing specifically on the user interface [ …]” . The goal is to ensure that the device user interface has been designed such that the user errors that could cause harm or degrade medical treatment are either eliminated or reduced to the extent possible [39].

Heuristic evaluation was the first of the usability evaluations undertaken during this study as its objective is to identify weaknesses in the design, especially when use error could lead to harm [39]. This review can be completed by usability experts, thus providing an opportunity to address major usability issues before the end-users interact with the user interface.

Usability issues were identified, listed, and then categorized by severity as minor, moderate, major, or catastrophic or “show-stoppers” by a human factors engineer; severity estimates were based on frequency, impact, and persistence of errors [40]. In addition, extensive quality assurance was also conducted by a member of the human factors engineering team using various clinical scenarios to confirm that the program produced the expected result.

The user interface was refined in response to the usability issues that were identified prior to proceeding to the Phase 4.

### Phase 4: usability testing

Cognitive task analysis [41] was conducted in 45-min sessions; users were asked to “think aloud” [42] as they performed specific tasks to cover the major functionalities of the SDM toolkit (clinician enabler, *MyDiabetesPlan*, and patient workbook).

#### Participants

A total of 11 patient-clinician dyads were invited to participate (3 usability cycles of 4, 4, and 3 dyads respectively). Clinicians were first recruited; each clinician then identified a patient with diabetes and 2 other comorbidities. These patients were invited by the research coordinator to participate in the usability testing. Research has shown that up to 80% of usability issues can be identified through 5 to 8 participants [41].

#### Data collection

A research team member with expertise in human factors engineering conducted each session in the primary care setting using the live website, a structured interview guide, and predefined task (completing the *MyDiabetesPlan*). The following data were documented: navigation choices, errors made, when and where they encountered confusion or frustration, task completion rate, and time spent on the PtDA toolkit or individual tasks within it. Participants were then interviewed regarding satisfaction, strengths and weaknesses of the toolkit, helpful/not helpful/missing content, the quality of decision support, and general comments. All interviews were audio- and video -recorded, and field notes were kept of all sessions as a further source of data.

#### Analysis

Data analysis was conducted as described in Phase 1.

Throughout Phases 1 to 4, *MyDiabetesPlan* was refined iteratively by study team members including a graphics designer and computer programmer.

## Results

### Phase 1: feasibility testing

#### Participants

Seventeen interviews were conducted (7 people with diabetes, 6 physicians, 2 nurses, 1 dietitian, 1 pharmacist). Clinical practice and sociodemographic characteristics are indicated in Additional file 1: Table S2.

#### Themes

We analyzed and reported clinician and patient interviews separately to better understand the unique experiences and perspectives of each party involved in the decision-making and goal-setting.

#### Themes from people living with diabetes

We identified the following themes from interviews with people living with diabetes; representative quotes are listed in Additional file 1: Table S3a:

1. Approaches to decision-making were diverse and dynamic:

Patient participants described a spectrum of decision-making approaches, including physician-led, IP and collaborative methods, and patient-led approaches. For example, some participants described being told what to do by their physician. Other participants described a more collaborative role for physicians, whereby the physician provided information and reassurance, as well as a collaborative IP team-based approach. Another group of participants described being the sole owner of the decision.

Participants reported a variety of factors that affected the approach they selected on a case-by-case basis, including the specific decision to be made (e.g. making a dietary change vs starting insulin), context of care (e.g. lack of continuity of care forces the patient to make their own decision), and patient preference (which is grounded in cultural and generational expectations). Despite describing a variety of decision-making approaches that they experienced, many participants stated a preference for a patient-centred “50:50” partnership, emphasizing the critical nature of patient engagement, with resultant empowerment, and ownership ultimately facilitating behaviour change.

(2) A trusting therapeutic relationship and dialog as critical precursors to SDM, the sum of which promotes patient empowerment (barriers and facilitators to SDM):

Participants enumerated many facilitators of SDM, which we categorized into patient factors and patient-clinician factors. Patient factors included assertiveness (i.e. patients educating clinicians about SDM), adequate knowledge about their condition (i.e. actively seeking health information resources), and tailored information regarding their own health (i.e. hard copy records). However, participants emphasized the central role of their relationship with the clinician, expressing not only the need for accessibility, comfort, and familiarity, but the critical importance of trust, listening, and dialog.

Conversely, the absence of these factors, specifically, lack of assertiveness and knowledge, and lack of accessibility and a therapeutic relationship, were identified as barriers to SDM. Additionally, participants reported competing illness as a barrier to decision-making and goal-setting, yet shared many strategies they used to cope with it, namely prioritization. Participants prioritized competing illness by acuity, severity, symptoms, and impact on their function.

(3) Goal-setting was a dynamic process, distinct from goal achievement:

Participants’ approach to goal-setting was a dynamic process, changing with life context and concurrent medical problems. Competing comorbidities, in particular, mental health, often acted as barriers to goal-setting. Similar to SDM, knowledge of one’s health as well as a therapeutic relationship with one’s healthcare team were facilitators of goal-setting.

Participants made a distinction between goal-setting and goal achievement, in some cases recognizing the disconnect between wanting to *attain* a certain identity (i.e. goal-setting) yet not willing to *perform* the necessary actions to get there (i.e. goal achievement). Others bridged this gap by identifying and implementing an action plan. A third group focused on action only, downgrading the importance of the outcome or goal as insignificant if it was not accompanied by an action plan.

#### Themes from clinician participants

We identified the following themes from interviews with clinicians; representative quotes are listed in Additional file 1: Table S3b:

1. Dual complementary approaches to the complex patient (holistic and disease-specific) were used (looking at the forest *and* the trees):

Clinician participants described two complementary approaches, one holistic and the other disease-specific; they sought to understand the patient’s life story as well as biopsychosocial context. This information was used to develop a discrete list of medical priorities. After considering concurrent comorbidities (e.g. mental health), life circumstances (e.g. paying mortgage) and the person’s agenda, *and* providing support, clinicians then identified priorities for management. Concerns were prioritized across a hierarchy of factors that characterized the patient’s condition: life- or safety-threatening (e.g. angina, depression), symptomatic, and acute or new. These issues were prioritized over those that were asymptomatic and chronic. After explaining interactions with other conditions, negotiation and compromise were required to achieve an individualized plan tailored to the patient. Other facilitators in caring for the complex patient included engagement of other team members, frequent disease-specific appointments, and multi-disease flowsheets.

2) Patient-provider agendas for goal-setting were often conflicting (barriers and facilitators to goal-setting):

Clinician participants struggled with goal-setting when there was a disconnect between goals set by the patient (including not setting any goals) and potential goals selected by themselves for the patient. For example, they expressed feelings of futility when patients repeatedly attended appointments with no progression in goal-setting or behaviour change. Similarly, they were frustrated even with engaged patients who selected priorities that were not congruent with what they as clinicians considered important. Strategies that they identified to help bridge these conflicting agendas include engaging the patient, delving into the patient’s perspective, providing the patient with more information, and involving the IP team. Facilitators of goal-setting included use of clinical practice guidelines as well as a goals flowsheet integrated into the electronic medical record.

3) A flexible approach to decision-making was needed:

Clinicians’ approaches to decision-making were closely tied to their professional identity. On one end of the decision-making spectrum, some clinicians saw themselves as educators, wherein their primary role was to provide information. On the other end of the spectrum, a few clinicians saw themselves as decision-makers, wherein their role was to make the decisions in some situations. In the middle were clinicians who saw themselves as consultants, who worked to understand the patient’s goals and perspectives, then provided options and some guidance as to the best option. These roles were fluid; the decision-making approach was tailored to the individual patient based on patient preference and patient education level. A few clinicians endorsed an IP team approach to decision-making that encompassed many of these roles. Clinicians emphasized the critical need to engage the patient because of the chronic nature of diabetes, and recognized that ultimately, the patient has the final say.

4) Conflicting clinician-patient plans can be resolved with SDM through active listening in a therapeutic relationship:

Clinicians recognized the value of SDM, and outlined facilitators, barriers, and their perspectives on the role of formal SDM tools. They emphasized that SDM enabled the active involvement of the patient in the decision-making process, thus increasing patient accountability, “adherence” to treatment recommendations and satisfaction. They noted the challenges presented by competing health concerns as well as conflicting clinician and patient agendas, and described struggling with the “patient making the wrong decision”. Notably they emphasized the importance of a longitudinal process and relationship, yet this requirement was at odds with the limited time of scheduled appointments.

Clinicians emphasized the critical importance of a longitudinal relationship with a supportive primary care physician who considered the patient as a whole, and who actively listened to the patient in an effort to understand the patient’s perspective. From a practical perspective, they also noted that a patient with the appropriate knowledge base was an essential requirement for SDM.

### Phase 2: *MyDiabetesPlan* and implementation strategy development

#### *MyDiabetesPlan* development

While clinical goals consist of cardiometabolic risk factor targets, we found in Phase 1 that patients’ goals were informed by their personal values and life contexts. Thus, we designed the tool to collect a detailed baseline profile containing each patient’s hobbies (specifically categorized as requiring the ability to see or move around), most feared complication (for example, dialysis, blindness, etc.), sexual activity level, barriers to diabetes management (for example, medication non-adherence), and social supports in addition to routine demographic and clinical data.

We used the baseline profile to help patients select a “goal”, which we conceptualized as a specific diabetes complication that a patient would want to avoid in their life (for example, avoiding blindness). We prepared a list of 8 goals, and instructed each patient using the tool to select one. Our testing indicated that the 8-item list was too complicated, so we narrowed the list down to the 3 most relevant goals by ranking each goal using a scoring algorithm. This scoring algorithm considered all relevant characteristics in the baseline profile, including both patient values and clinical data. For example, a patient with a history of diabetic retinopathy might fear having a stroke, and also enjoy marathon running as a hobby. Their history of retinopathy would add 1 point towards the goal of “avoiding blindness”. Additionally, their fear of stroke and enjoyment of marathon running (which requires preserved motor function) would each add 4 points, with a total of 8 points for the goal of “avoiding stroke”. In this simplified example, avoiding stroke (8 points) would be ranked above avoiding retinopathy (1 point). We weighted the point value of clinical risk factors (e.g. age, smoking, retinopathy, etc.) based on published risk-prediction algorithms from large studies [31–38]. We weighted patient-important factors more strongly than clinical risk factors because of the patient-centred focus of this tool. We adjusted the value of all weights according to our findings from extensive iterative testing with patients and interprofessional clinical experts. To contextualize the selected goal, we further asked each patient using the tool to identify their motivation for achieving this goal (within a free-form text box) according to his or her personal values, hobbies, and lifestyle. For example, a patient could select a goal as “I want to avoid blindness”, and identify their motivation as “because I want to continue to see my grandchildren”.

To develop goals into action plans, we created an extensive set of “options”, conceptualized as specific and measurable tasks that patients could do to help them achieve their selected goal. For example, one option could be “taking my metformin in the morning and evening at least 6 days a week”. Because there were > 100 possible options, we again narrowed the list to the 3 most relevant options for each patient using a scoring algorithm. Similar to the process described above, patients using the tool were then instructed to select their preferred options from this shortened list. The algorithm accounted for both patient-important and clinical factors, with patient-important factors again being weighted more strongly. We further adjusted the values of these weights based on the iterative development process.

As such, by enquiring about and eliciting patient-important values, beliefs, facilitators, and barriers to care, *MyDiabetesPlan* was designed to enable dialog between patients and their clinicians and thus facilitate cultivation of a therapeutic relationship and shared decision-making.

#### Implementation strategy development

To develop an implementation strategy, we compiled barriers and facilitators to goal-setting, SDM and decision aid use in the care of people with diabetes with multiple comorbidities, from the literature [43, 44], as well as results from Phase 1. We categorized barriers and facilitators into systems-related, clinician-related and patient-related factors, and then outlined potential strategies to overcome the barriers to include as components of our implementation strategy (Table 1). For example, to tackle lack of organizational commitment, we identified a clinical champion in a leadership position (e.g. site investigator) and engaged each site via team and individual meetings, and designed the tool to optimize its integration into usual care. In addition, to support the training of clinicians in using *MyDiabetesPlan*, we developed an online video and a concise handout that emphasized the nine essential elements of SDM in the context of *MyDiabetesPlan* [45]. In addition, we created a training manual for research team members to use with clinicians, which provided step-by-step instructions of how *MyDiabetesPlan* is used and how to integrate it into practice; research team members met with each participating clinician for a 60-min training session, created bookmarks and links on their clinic computer, provided a practice exercise, and followed up one week after the training session.

**Table 1:** Development and Refinement of *MyDiabetesPlan* and its implementation plan based on identified barriers and facilitators from literature and interviews

Level	Barrier	Facilitator	Component of implementation strategy
From Elwyn 2013 review – barriers and facilitators to PtDA implementation			
HCP	HCP attitude		HCP engagement throughout process including tool development Training session Training video Enabler card (laminated double-sided FAQ sheet)
	Lack of training	Training	
	Lack of trust, agreement with content of decision aid		
	Lack of time		Interprofessional involvement
	Not their role to identify patient		EMR prompt to use tool
Systems	Lack of organizational commitment	Clinical champion, in leadership position	Site investigator +/−other Engagement of FHT throughout process Integrated into usual care
		Systems level process supporting patient identification	EMR prompt to use tool
From Legare 2014 review – barriers and facilitators to SDM			
Systems		Barrier assessment	Done via feasibility testing (see below) Done at each site by developing process map with clinical team
Provider-directed		Educational meeting/outreach	Training session Practice session
		Distribution of education material	Training video Enabler card
		Audit & feedback	Review whether tool used Review how tools used Follow up phone call (clinical champion)
		Reminders	EMR Email
Patient-directed		Paper/internet DA Pamphlet	PtDA Training video Enabler card
Both		Above	
From Phase 1 feasibility data			
Systems	Lack of organizational commitment		See above
HCP	Competing health concerns		See below under patient
	Lack of time		Scheduled into dedicated visit Assessed in usability Interprofessional approach
	Inadequate patient knowledge		See below under patient
	Loss of interaction with patient		See below under patient
	Complexity		Assessed in usability Training
	Length		Assessed in usability Warn that first time is longer +/− prefill depending on use
		Flow sheet	Plan to be pasted into EMR medical note and made searchable
		Note from other HCP in EMR	
		Engaged team	Engagement of FHT throughout process Train all team members
Patient	Inadequate knowledge about own health	Dialog, listening	Use with HCP in first phase During HCP user training, emphasize role of tool in enabling dialog, importance of listening
	Inadequate knowledge about disease		Tool as springboard for discussion Tool provides information about disease management options
	Denial		Addressed in tool under health beliefs section
	Competing health concerns		Scheduled to use during dedicated diabetes visit
	Competing life issues		Acknowledged in initial screening phase of tool

*HCP* Healthcare professional, *FAQ* Frequently-asked questions, *EMR* Electronic medical record, *FHT* Family health team

### Phase 3: heuristic evaluation

The heuristic evaluation was completed from the perspectives of patients and revealed that the website met the following usability requirements: used straightforward language and clear instructions; provided a flow that assisted the patients in navigating the multi-step process; used sans-serif font for the body of the text (which is well-suited for online reading) and sufficient contrast on all sections of the website (as per Canadian National Institute for the Blind standard for legibility) [46]; used patient-centric text without clinical jargon except where necessary (e.g. medication names). However, the flow of the recommendation process was perceived to be complex, representing multiple steps that were embedded in higher level multiple steps. Based on this feedback, we redesigned the tool to follow the structure commonly used for online assessment tools, which consists of a data collection stage and the recommendation stage. We made minor changes to formatting and wording to further optimize comprehension and ease of use.

### Phase 4: usability testing

#### Participants

We conducted 11 sessions with various dyads (nurse and patient (*n* = 6), dietitian and patient (*n* = 2), physician and patient (*n* = 3)). Six of these dyads were paired to mimic real life use (i.e. patient first with nurse, then the same patient with the physician).

#### Usability errors

No critical issues were identified. However, several usability issues of “moderate” and “high” severity were identified, such as website navigation, clarity of terminology (e.g. who is considered a “health care provider”), and phrasing of questions (e.g. avoid double negative in answer choice) (Additional file 1: Table S4). Subsequent redesign and iterative testing demonstrated resolution of these issues.

### Refinement of *MyDiabetesPlan* and implementation strategy

Based on findings from our interviews and usability testing, we refined *MyDiabetesPlan* (see Additional file 2 for screenshots), as outlined in Table 1. In addition, to overcome implementation barriers identified in the literature and in our interviews, we developed an implementation strategy consisting of a training video, training session, enabler card, engagement of a clinical champion, and integration of *MyDiabetesPlan* output into the electronic medical record (Table 1). The training session and training videos emphasized the importance of dialog and therapeutic relationship as facilitators of shared decision-making.

## Discussion

Following principles of user-centred design [47] and adhering to IPDAS criteria [21], we developed a diabetes-focused, goal-setting PtDA to facilitate SDM by interprofessional teams, as well as a strategy with which to implement it in clinical practice. Working with patients, clinicians, and a human factors engineer, we found that people living with diabetes used diverse approaches to decision-making with a preference for SDM. Dialog and a trusting relationship with their clinicians were vital prerequisites to SDM, which together promoted patient empowerment. They viewed goal-setting as a dynamic process, distinct from goal achievement. Clinicians working with complex patients employed both holistic and disease-specific approaches in order to prioritize concerns through negotiation and compromise and achieve a tailored plan. They expressed frustration when patient priorities were discordant with their own, but sought to bridge this disconnect by further eliciting the patients’ perspectives. Clinicians’ approaches to decision-making were closely tied to their professional identity, ranging from that of an educator to that of a decision-maker. Though the approach they used was tailored to the individual patient, they recognized that the patient had the final say. While they also recognized the critical importance of SDM, clinicians highlighted again the challenge of conflicting clinician and patient agendas, the resolution of which depended on an appropriate knowledge-base on the part of the patient, and active listening on the part of the clinician, all within the context of a longitudinal relationship.

Both patients and clinicians reported “disconnects” between goal-setting and goal achievement, and “discordant” agendas, as challenges to SDM. Our finding regarding the disconnect between *attaining* a certain identity (i.e. goal-setting) yet not *performing* the necessary actions to get there (i.e. goal achievement) highlights the important objective of any decision-making process – that of reaching a compatible “middle” ground of congruence, mirroring the process described by Image Theory [48]. Image Theory postulates that individuals base their decision on three images: value image (their principles or “be” goals), trajectory image (their agenda or “do” goals) and finally, strategic image (their action plan). Potential approaches to bridging this gap include incorporating Value Theory-based strategies into the decision-making process such as compatibility testing (screening out incompatible options), followed by probability testing (deliberating between the remaining options) [49]. We integrated these processes into *MyDiabetesPlan*. In addition, we incorporated specific strategies cited by our participants to bridge this gap, such as action planning. This finding validates previous studies [48] and highlights the critical importance of including a clear action plan as part of the SDM toolkit.

Our finding regarding conflicting patient-clinician agendas, moderated by negotiation and compromise, highlights a fundamental philosophical issue regarding roles of patients and clinicians in decision-making. In SDM, the patients are the authorities on their own experience of living with an illness, the burden of disease on their lives, and how treatment plans may best suit their needs, while the physician is the expert in the evidence-based medicine and evolution of disease [50]. By its very definition, in the paradigm of SDM, the decision is a shared responsibility of both the patient and the physician, albeit with different responsibilities: one contributing expertise for themselves, and the other contributing expertise for the disease. When conflict arises between these priorities, SDM seeks to resolve this through deliberation to reach a common understanding [30], echoing the comments of our participants. In this way, the conflict between patient and physician autonomy can be resolved, with the anticipated outcome of patient beneficence. However, what happens in practice? The literature suggests a spectrum of behavior. On one end of the spectrum, physicians report an intent to use SDM, followed by its application in practice. However, they often employed subversive tactics to steer both patient and family towards the decision they perceived as correct [51]. On the other end of the spectrum, a questionnaire-based study found that nearly half (47%) of patients preferred the clinicians to make the decision without their participation and only 19% wished to share the decision equally with their physician, with 3% wishing to make the decision independently [52]. A systemic review of patient decision role preferences identified 115 eligible studies, which demonstrated increasing preference for shared decision-making with time: the majority of respondents preferred shared decision-making in 50% of the studies conducted before 2000, increasing to 71% of studies conducted after 2000 [53]. This is corroborated in a qualitative study exploring 51 patients’ preferences for decision-making approaches, which found that while some patients preferred full engagement in treatment decision-making, others preferred partial or minimal involvement, deferring to their physician due to clinical expertise and a trusting relationship [54]. Our findings – from both patients and clinicians – reflect this diversity of preference when describing their approach to decision-making, suggesting that a flexibility of decision-making approaches is an important facilitator of patient-centred care; that is, patient-centredness does not mean sharing all decisions, but rather taking into account and responding to the patient’s desire for sharing decision-making [55]. Together, these findings underline the importance of training clinicians on tailoring to patients’ needs and preferences, presenting information in an unbiased manner, empowering patients with knowledge about their disease, and listening and learning about the patient perspective, in our decision aid in order to bridge potential gaps between patient and clinician agendas.

Our study’s strengths include the systematic development process, as well as rigorous qualitative methodology. We employed evidence-based and user-centered development and implementation, which was facilitated by an interdisciplinary team and early engagement of knowledge-users. The team included expertise in shared decision-making, knowledge translation, information technology, primary diabetes care, and qualitative and quantitative research methods, as well as key knowledge users – primary care providers and people with diabetes. We ensured analytic rigour through the use of at least two coders, interview-by-interview validation, as well as field notes and meeting minutes documenting analytic decisions.

Study limitations included potential volunteer and recruitment bias.

## Conclusion

We developed a diabetes-focused, goal-setting patient-decision aid to facilitate SDM by interprofessional teams, as well as a strategy with which to implement it in clinical practice. We found that people living with diabetes used diverse approaches to decision-making with a preference for SDM. Dialog and a trusting relationship with their clinicians were vital prerequisites to SDM, which together promoted patient empowerment. Patients viewed goal-setting as a dynamic process, distinct from goal achievement. Clinicians working with complex patients employed both holistic and disease-specific approaches in order to prioritize concerns through negotiation and compromise and achieve a tailored plan. Clinicians’ approaches to decision-making were closely tied to their professional identity, ranging from that of an educator to that of a decision-maker. Together, these findings underline the importance of training clinicians on tailoring to patients’ needs and preferences, presenting information in an unbiased manner, empowering patients with knowledge about their disease, and listening and learning about the patient perspective, in our decision aid in order to bridge potential gaps between patient and clinician agendas. While barriers exist to the successful implementation of decision aids into clinical practice [21], we hope that integrating findings from this study into decision aid development and implementation will optimize uptake into clinical care and thus, improve decision quality.

## Additional files

Additional file 1: **Table S1.** COREQ checklist. IPDAS checklist. Feasibility interview guide. **Table S2.** Demographic characteristics of interview participants. **Table S3.** Representative quotes from feasibility interviews. **Table S4.** Usability issues. (DOCX 507 kb)

Additional file 2: Screenshots of *MyDiabetesPlan*. (DOCX 803 kb)

## Data Availability

See Additional Files.

## Abbreviations

IP: Interprofessional
KTA: Knowledge to Action
PtDAs: Patient decision aids
SDM: Shared decision making
