# Supplementary material for: Beyond pros and cons – developing a patient decision aid to cultivate dialog to build relationships: insights from a qualitative study and decision aid development
Source: BMC Med Inform Decis Mak. 2019 Sep 18;19:186. doi: 10.1186/s12911-019-0898-5 (PMC6749701; doi:10.1186/s12911-019-0898-5)
Supplement: Supplementary file 1 — Table S1. COREQ checklist. IPDAS checklist. Feasibility interview guide. Table S2. Demographic characteristics of interview participants. Table S3. Representative quotes from feasibility interviews. Table S4. Usability issues. (DOCX 507 kb) [file 12911_2019_898_MOESM1_ESM.docx]

**Additional File 1**

**Table of contents**

1. [**Table 1: COREQ checklist**](#T1)
2. [**IPDAS checklist**](#T2)
3. [**Feasibility interview guide**](#T3)
4. [**Table 2: Demographic characteristics of interview participants**](#T4)
5. [**Table 3: Representative quotes from feasibility interviews**](#T5)
6. [**Table 4: Usability issues**](#T6)

**1)** **Table 1:** **COREQ checklist**

| **No. Item** | **Guide questions/description** | **Reported on Page #** |
| --- | --- | --- |
| **Domain 1: Research team and reﬂexivity** |  |  |
| *Personal Characteristics* |  |  |
| 1. Inter viewer/facilitator | Which author/s conducted the interview or focus group? | Methods (7,8) |
| 2. Credentials | What were the researcher’s credentials? E.g. PhD, MD | Methods (1,5, 7,8) |
| 3. Occupation | What was their occupation at the time of the study? | Page 5 |
| 4. Gender | Was the researcher male or female? | N/A |
| 5. Experience and training | What experience or training did the researcher have? | Methods (7) |
| *Relationship with participants* |  |  |
| 6. Relationship established | Was a relationship established prior to study commencement? | Methods (Page 5 -11) |
| 7. Participant knowledge of the interviewer | What did the participants know about the researcher? e.g. personal goals, reasons for doing the research | Methods (7,11) |
| 8. Interviewer characteristics | What characteristics were reported about the inter viewer/facilitator? e.g. Bias, assumptions, reasons and interests in the research topic | Methods (7,11) |
| **Domain 2: study design** |  |  |
| *Theoretical framework* |  |  |
| 9. Methodological orientation and Theory | What methodological orientation was stated to underpin the study? e.g. grounded theory, discourse analysis, ethnography, phenomenology, content analysis | Methods (5) |
| *Participant selection* |  |  |
| 10. Sampling | How were participants selected? e.g. purposive, convenience, consecutive, snowball | Methods (6) |
| 11. Method of approach | How were participants approached? e.g. face-to-face, telephone, mail, email | Methods (5, 6) |
| 12. Sample size | How many participants were in the study? | Results (11) |
| 13. Non-participation | How many people refused to participate or dropped out? Reasons? | N/A |
| *Setting* |  |  |
| 14. Setting of data collection | Where was the data collected? e.g. home, clinic, workplace | Methods (7,11) |
| 15. Presence of non-participants | Was anyone else present besides the participants and researchers? | Results (12,17) |
| 16. Description of sample | What are the important characteristics of the sample? e.g. demographic data, date | Results (12) |
| *Data collection* |  |  |
| 17. Interview guide | Were questions, prompts, guides provided by the authors? Was it pilot tested? | Methods (7) |
| 18. Repeat interviews | Were repeat inter views carried out? If yes, how many? | N/A |
| 19. Audio/visual recording | Did the research use audio or visual recording to collect the data? | Methods (7,11) |
| 20. Field notes | Were ﬁeld notes made during and/or after the interview or focus group? | Methods (7,11) |
| 21. Duration | What was the duration of the inter views or focus group? | Methods (7,11) |
| 22. Data saturation | Was data saturation discussed? | Methods (11) |
| 23. Transcripts returned | Were transcripts returned to participants for comment and/or correction? | N/A |
| **Domain 3: analysis and ﬁndings** |  |  |
| *Data analysis* |  |  |
| 24. Number of data coders | How many data coders coded the data? | Methods (8,20) |
| 25. Description of the coding tree | Did authors provide a description of the coding tree? | N/A |
| 26. Derivation of themes | Were themes identiﬁed in advance or derived from the data? | Methods/Results (8,12) |
| 27. Software | What software, if applicable, was used to manage the data? | Methods (8) |
| 28. Participant checking | Did participants provide feedback on the ﬁndings? | Page 10, 17 |
| *Reporting* |  |  |
| 29. Quotations presented | Were participant quotations presented to illustrate the themes/ﬁndings? Was each quotation identiﬁed? e.g. participant number | Results (12,14) |
| 30. Data and ﬁndings consistent | Was there consistency between the data presented and the ﬁndings? | Relationship to existing knowledge |
| 31. Clarity of major themes | Were major themes clearly presented in the ﬁndings? | Results (12,14) |
| 32. Clarity of minor themes | Is there a description of diverse cases or discussion of minor themes? | Discussion (18-20) |


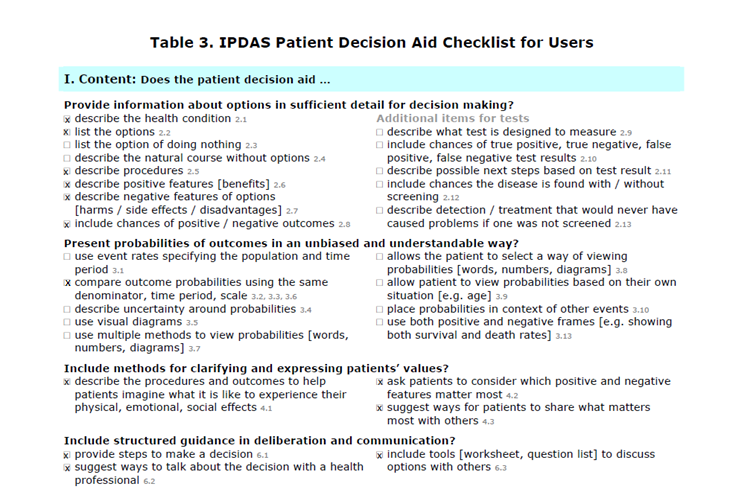
2) **IPDAS checklist**


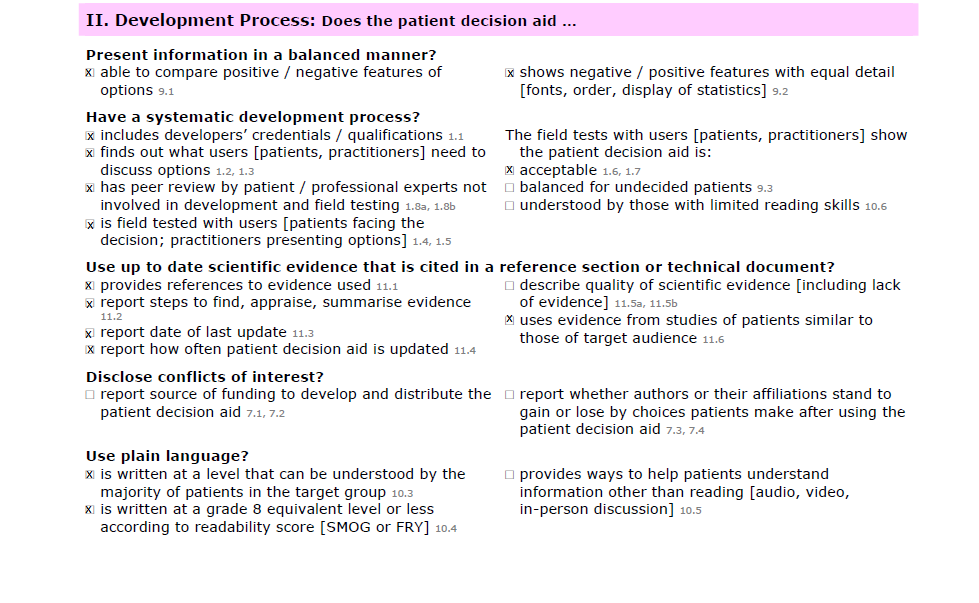


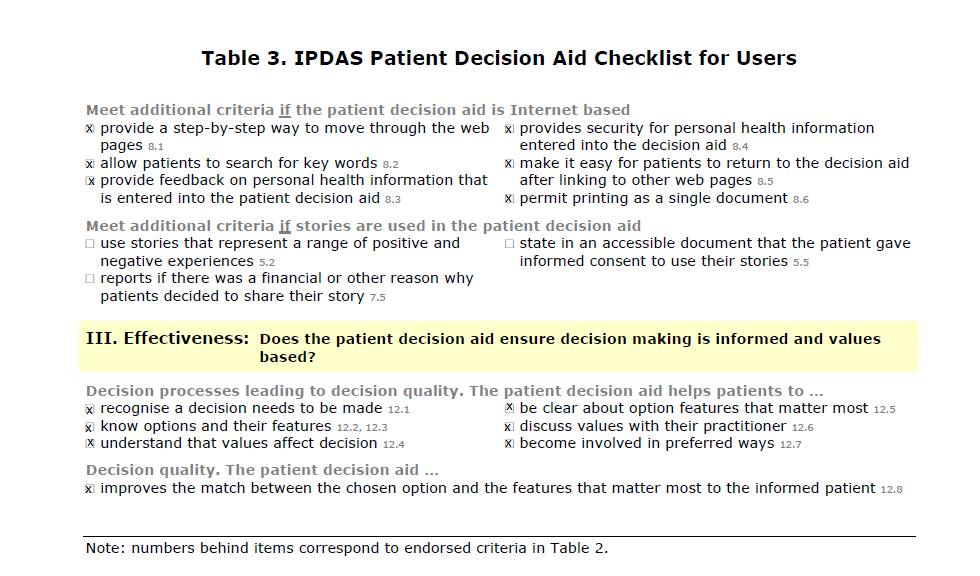


3) **Feasibility interview guide**

Thank you for taking the time to meet with me today. My name is_____________ and I work with Dr. Catherine Yu from St. Michael’s Hospital (*and other docs from Sunnybrook etc).* We are conducting interviews with people who have diabetes along with other chronic diseases to help us understand how you prioritize your health care needs for managing diabetes in the context of having other chronic diseases. We are also interviewing health care professionals.

Specifically we will discuss how you make decisions about your personal health care. The goal of this research project is to increase the involvement of patients in decisions about their health care. Findings from these interviews will help us design an aid to help patients prioritize their health care goals and make decisions about their diabetes.

The interview should take about 45 to 60 minutes. I will be taping the interview so that I don’t miss any of your comments. All of your comments will remain confidential. You don’t have to respond to any question that makes you uncomfortable.

Have you had a chance to read the consent form? Do you have any questions about the consent or about what I have just explained to you? Are you willing to participate?

Great, let’s start.

1. In your experience, how are health care decisions made? (e.g. decisions are made along with a health care professional, a health care professional makes the decision, the health care professional offers options and allows the patient to make the decision)

PROBES (based on response)

- Can you describe a time when you made a health care decision along with your HCP
- Can you describe a time when a health care professional made the decision?
- Can you describe a time when you made a health care decision on your own
- Can you describe a time when a HCP offered options and allowed you to make a decision
- How did any of the above experiences make you feel?

1. In your opinion, how **should** health care decisions be made? How would *you* like decisions to be made? (e.g. decisions are made along with a health care professional, a health care professional makes the decision, the health care professional offers options and allows the patient to make the decision)

PROBES (based on response)

- If you feel that health care decisions should be made along with a health care professional, how involved do you think you should be in the decision? Describe how comfortable or uncomfortable you might feel having a discussion about your health care with your HCP?
- If you feel that the HCP should offer advice and allow you to make the decision, how much information is enough? What resources might you use besides your family doctor or another HCP from your doctor’s practice to help you make a decision?
- If you feel that the HCP should make the decision on your behalf, please tell me why this makes you feel comfortable.

1. I’d like to talk to you about setting goals for your health care. Suppose you were asked, “what are your health care goals?” How would you respond?

PROBES (based on response)

- If you’ve never thought about health care goals and you were asked to set goals for yourself, how would you go about setting goals?
- You’ve got some concrete goals – how did you decide on those goals?
- Why did you choose that/these goals in particular? (why are these goals important to you?)
- How confident are you that you will achieve these goals?
- How will you achieve these goals?
- If achieved, how will this goal affect/change your life or your health?

1. Let’s talk about what influences the choices you make when setting goals for your overall health and your health care.
   - Let’s start by asking: what health care goals are important to you? E.g. staying pain-free, living a long life, keeping good vision etc.
   - How did you make that choice?
     - First of all, does it matter? Does setting goals for your health care and the choices you make actually make a difference? [perceived behavioural control]
   - Does it matter what other people think? Who influences your personal decisions about your health and health care? [perceived norms]
     - PROBES –your family, your physician, another HCP, friends, experts offering advice on TV, social media, the Internet
   - What information is necessary to make decisions about your health care and where do you find this information?
   - When you think about setting your health care goals, what emotions do you feel? How do emotions shape or influence the choices you make or goals you select? [attitude] What do you need from an emotional standpoint to help you make this decision?
2. You have diabetes and at least one additional chronic disease. Let’s talk about how you balance your health care and make decisions about treating multiple diseases.

- How do you decide which disease treatments take priority?
- People with diabetes and other chronic disease have many different behavior/lifestyle changes to make. What behaviour/lifestyle modifications have you made and how did you decide to make these changes?

1. Can you think of a time when a health care professional supplied you with any type of an aid to help you make a decision regarding prioritization of your health care goals and treatments?

We are now going to ask you to work through a prototype of an aid that will help you set goals and strategies for managing your diabetes. I would ask you to speak out loud and express your thought processes and ideas as you are going through the exercise.

Prompts: we completed this aid online in an electronic format (or, if in person, on a paper workbook). Would you prefer a different format?

Should this aid be completed before your appointment with your health care professional or during your meeting with your HCP? Why?

How do you feel about the way the information in the aid was presented to you? Should there be more/different questions? More examples/case studies

How relevant are the different sections? Goals, strategies etc.

How would this aid help you make decisions about goals and strategies you’re your health care?

**4)** **Table 2: Demographic characteristics of interview participants**

| **Health care providers (n=10)** | | |
| --- | --- | --- |
| **Gender** | | |
|  | Male | 1 |
|  | Female | 9 |
| **Years in Practice** | | |
|  | < 5 year | 6 |
|  | 5-9 year | 1 |
|  | 10-14 year | 1 |
|  | 15-20 year | 1 |
|  | >20 year | 1 |
| **Profession** | | |
|  | MD | 6 |
|  | RN | 2 |
|  | RD | 1 |
|  | Pharm | 1 |
| **CDE** | | |
|  | Yes | 3 |
|  | No | 7 |
| **Remunerate** | | |
|  | Salary | 7 |
|  | Fee for Service | 1 |
|  | Combination | 2 |
| **Setting** | | |
|  | Academic | 5 |
|  | Community | 3 |
|  | Both | 2 |

| **Patients with Diabetes (n=7)** | | |
| --- | --- | --- |
| **Gender** | | |
|  | Male | 2 |
|  | Female | 5 |
| **Age** | | |
|  | 40 – 59 years | 3 |
|  | 60 – 79 years | 4 |
| **Years with Diabetes** | | |
|  | Less than 5 years | 2 |
|  | 5 to 9 years | 4 |
|  | More than 20 years | 1 |
| **Type of Diabetes** | | |
|  | Type 1 | 0 |
|  | Type 2 | 7 |
| **Taking Insulin** | | |
|  | No | 7 |
|  | Yes | 0 |
| **Co-Morbidities** | | |
|  | Any kind of heart disease | 3 |
|  | Stroke | 1 |
|  | High Blood Pressure | 6 |
|  | Kidney Disease | 2 |
|  | Nerve Disease | 1 |
|  | **Cancer** | |
|  | Renal Cell Carcinoma | 1 |
|  | Breast Cancer, Bladder Cancer | 1 |
|  | Melanoma | 1 |
|  | Arthritis | 2 |
|  | Inflammatory Bowel Disease | 1 |
|  | **Other** | |
|  | Major Depression | 1 |
|  | Conn Disease | 1 |
|  | Underactive Thyroid | 1 |
|  | Sciatica and Cholesterol | 1 |
| **Education** | | |
|  | High School | 1 |
|  | College | 3 |
|  | University | 3 |
| **Annual Income** | | |
|  | < $15, 000 | 1 |
|  | $15, 000 to $29, 999 | 1 |
|  | $30, 000 to $59, 999 | 2 |
|  | $60, 000 to $89, 999 | 3 |

**Table 3a: Representative quotes from feasibility interviews with people living with diabetes**

| **Theme** | **Subtheme** | **Quote** |
| --- | --- | --- |
| **Approach to decision-making** | Physician telling patient decision | At that point I was told, well you are going to be put on metformin and you have to lose weight and I think that was pretty much it. No, no, no, I was the patient. [I was told] Here is your metformin and lose weight... well, diet and exercise... I mean, I had that message thrown at me all the time but my mind was somewhere else. *[1A14]* |
|  | Physician providing information and support | I don’t have enough knowledge to be able to um, [Um hmm] say oh no, that’s not it. So, this is [Um hmm] where the doctor needs to be able to I guess maybe get your find out what your fears are and, and [Um hmm] reassure you. *[1A17]* |
|  | Collaborative, team-based approach | So, they’re, they’re learning as much as you’re learning [Um hmm] I, I got told that years ago how, work with the doctor you are working with. [Okay] Because really you are working as a, you’re working as a team. So, I’ve always had that concept with whosever on my team [Um hmm] um, sometimes [Okay] it’s takes time to see what’s happening. [Um hmm] Um, and if, if you hit a road block we’re, we’re all in this together right? *[1A16]*  Here there is dialogue, here’s the numbers, the blood works done … look it they show you the graph. There is interaction, you are allowed to see the information on your files as much as you need to. So it is a collaborative here that they use doctor physician. So we would interact, sometimes they would have a nurse doing it, depending on their man power here I guess. So it’s more in discussion… *[1A11]* |
|  | Patient as sole owner of decision | And they can suggest and advise but that doesn’t mean that I am going to do it… It really is up to me. *[1A18]* |
| Factors affecting decision-making approach | Patient preference, grounded in cultural and generational expectations | Because he came from the old country, Greece where the doctor was God and the patients were just sheep. He still had that attitude. At the beginning of our marriage, he was going to Greek doctors because he felt more comfortable speaking in their language and I knew their attitude and I kept saying, don’t go there, you don’t know what’s going on, quit the doctor. The idea of changing a doctor was just foreign to him. *[1A14]* |
| Preference for SDM |  | One of the reasons why joined the clinic and she herself takes the time to sit there and listen if I have any issues and watches and sit there and says, 3 years ago this was your A1C. It says here now what can you do to do about it and we do, we discuss the medications and she would like to eventually take me off one of the medications. Initially she was going to take me off metformin and I thought, metformin is a fairly good drug actually. So why don’t we go with the other one that I’m on? And she said yeah, you’re right, let’s do that instead, we will focus on that. So there is a conversation that goes on between her and I. *[1A14]* |
|  | Critical importance of patient engagement | Whereas with me, if the doctor is not engaging me in my health care then it’s time for me to go. *[1A14]* |
|  | Patient takes ownership | Yes, I should absolutely be involved, 100% this is my life, my body 7/24 nobody else’s. So I have found that there has been times and actually I caught my family doctor doing this, she had an intern and he picked up something on the heart and I know it’s there anyways and she almost scolded him and I’m going I know what it is...you know. Give the patient the benefit of the doubt …and I think it is so important to be part of the decision making process. The process is, I go to the doctor because I have a problem, the doctor does not come to me and then based on that, do some tests, found out yes I agree I will have this blood work done. I won’t forget it, I’ll make it a priority and then once we have the facts a follow up of the facts, how do they relate and from the appointment, I take the information and I process it to say, what do I need to do in my life to accommodate what I need to do. So it always belongs to me, I never ever, ever , ever, ever , ever just walk in blindly here I am do whatever you want. No, no, no. *[1A11]* |
|  | Facilitate behaviour change | I would like to have some input in it. I’m probably not going to resists as much if I have some input in it. You know, just like a kid, if you are told to do something, forget that... you know... give me the pros and cons, so then yes, make it feel like I’m taking the decision *[1A15]* |
| **Facilitators of SDM** | Assertiveness | But I’d like to think of myself as fairly well educated and I do have an assertive nature toward my health care and I’ve told her when I first came on the clinic and said look, the way I look at my health care is as a 3 prong stool . There is you, there is me and I also put in naturopathy as well. And I said, those are the 3 corner stones, not one is super greater than the other, they are all part of the whole thing. And if everybody does their thing then I will benefit. *[1A14]* |
| Patient factors | Adequate knowledge about their condition | I see my doctor every 3 months. Usually not much more often than that unless I have an issue. You know, using his guidance and seeking help when I need it from different sources. Could be the internet, could be talking to my pharmacist... any of those because they are pretty good too. *[1A15]* |
|  | Tailored information regarding their own health | That’s who I am oh, it’s going down and [Yeah] now they have the charts. [Um hmm] That’s very empowering to me [Okay] to see my sugars going down [Sure] to a very healthy level [Um hmm] and it may get to be like the blood pressure where you’ve got to watch it [Um hmm] you know, like but like this month it’s like your system is shifting [Um hmm] so, so you, you have to be on top of it [Um hmm, okay] so. *[1A16]* |
| Patient-clinician factors | Accessibility | I think the other key piece is access to my physician.  Very important [Um hmm] you know, I, I don’t want to see them 3 months from now {Laughing} if I need to see them [Um hmm, of course] so you know, access is very important. *[1A18]* |
|  | Trust | Here you have many things that you are focusing on [Yeah] so to me my, my doctor has really been my life saver because [Yeah] you, something has to keep you steady [Yeah] um, stable.  It just gave you full confidence that you were going to make, make it through a tough time.  And that I let them know I could trust them [Um hmm] and they also let me know they, they were trusting me because you had/have to be really in tune with yourself [Um hmm] for and communicate with that doctor for that doctor to be able to help you [Yes], you have to be so in tune with all of the little things. *[1A16]* |
|  | Listening, dialog | One of the reasons why joined the clinic and she herself takes the time to sit there and listen if I have any issues and watches and sit there and says, 3 years ago this was your A1C. It says here now what can you do to do about it and we do, we discuss the medications and she would like to eventually take me off one of the medications. Initially she was going to take me off metformin and I thought, metformin is a fairly good drug actually. So why don’t we go with the other one that I’m on? And she said yeah, you’re right, let’s do that instead, we will focus on that. So there is a conversation that goes on between her and I. *[1A14]* |
| **Barrier to SDM** | Competing illness | But if it’s a chronic thing, it competes with the diabetes and often times I walked away from the appointment not feeling that it were thorough enough. *[1A11]* |
|  | Approach to competing illness - prioritization | We can’t deal with 5 things at one time [Okay] pick one or two keep it simple conquer it and then book another appointment next time. [Okay] So, I think in 3 years I saw the doctor every month. [Okay] So, I had a pretty steady support but I was chronically sick. *[1A16]* |
|  | Prioritization by: acuity | Take medications, that’s what I needed... there is some of them the medication for high blood pressure to me isn’t as important as these 2 other pills that keep me out of the hospital and I no longer feel like killing myself and that was one of the things that was going on. Just not because of stress or anything else, just the body triggered. *[1A11]* |
|  | Severity | Oh yeah, [Okay] because I have been sitting at like 200 over you know, high, high numbers [Yes] like and you feel terrible [Yeah, I bet] it’s a terrible feeling. So, hypertension is actually been more prevalent in my mind lately [Yeah] than the depression or the kidney cancer right? *[1A18]* |
|  | Symptoms | That’s hypothetical and then the high blood pressure doesn’t seem to… It’s there and it doesn’t seem to… every once in a while I can feel it, but very, very seldom. If I’m out of sorts with the diabetes I know it right away. *[1A12]* |
|  | Impact on function | .. It was the shock in 2 and a half months, to going from being a full functioning working everyday doing all that I wanted to do male not be able to walk in a straight line without a walking stick. *[1A12]* |
| **Goal setting** | Dynamic | I think this year my main focus is a little different, I’ve worked really hard at eating healthy the next one is getting that daily walking. *[1A16]* |
|  | Mental health as barrier to goal setting | There is lots of days you just say the heck with it, I’m just sick and tired and taking of poking at myself and taking these x number of pills. I’m just going to enjoy my life and if I die sooner well it’s a few years less in a nursing home. Yeah, there is no question there is the up and down moods that come in there. *[1A14]* |
|  | Facilitator: access to interprofessional care team | I think the other key piece is access to my physician. Very important you know, I, I don’t want to see them 3 months from now if I need to see them so you know, access is very important. And that’s why this [healthcare institution] is just the only way I can put it is I felt like I was like in a pot of gold when I found this place… Well, I you know, I, I’ve seen, they have a dietician they have a pharmacist you know, they have all of the supports right here so it’s not a matter of well I will refer you to and you wait 6 months to see it’s right here. *[1A18]* |
|  | Facilitator: knowledge of one’s one health | That’s who I am oh, it’s going down and [Yeah] now they have the charts. [Um hmm] That’s very empowering to me [Okay] to see my sugars going down [Sure] to a very healthy level [Um hmm] and it may get to be like the blood pressure where you’ve got to watch it [Um hmm] you know, like but like this month it’s like your system is shifting [Um hmm] so, so you, you have to be on top of it [Um hmm, okay] so. *[1A16]* |
|  | Goal setting vs goal achievement | I have set goals, whether they are being met is another story. *[1A14]* |
|  | “Be” goal vs “Do” goal | But I can still be if I choose, I can still be an active, physically active person. And, and it’s a matter of choosing to do so. *[1A17]* |
|  | Goal achievement requires action plan | Goals is not you know, you may have a goal that you are aiming, striving for, but it’s the plan of the journey, how you’re going to get to that goal that’s really important. *[1A18]* |
|  | Insignificance of goal | It you know, you, you, I mean there’s no sense in setting a goal if you have no way idea in the world of how you are going to get there. *[1A16]* |

**Table 3b: Representative quotes from feasibility interviews with clinicians**

| **Theme** | **Subtheme** | **Quote** |
| --- | --- | --- |
| Complementary approach to complex patient | Holistic approach | It comes with mental health. It comes with stresses in… in their personal lives. You know often when they’re diagnosed they’ve got… they’ve got a big stress in their lives [inaudible 00:03:11] You know a new cancer diagnosis and they’re on Prednisone or… or there’s other stuff going on. You know they’re worried about their kids or their mortgages or whatever.  It’s… it’s…diabetes is not just in isolation. Its… it’s the whole… the whole picture. The whole patient profile. *[1A01]* |
|  | Disease-specific: | |
|  | Prioritization by threat to life or safety | So the things I tend to look at are, what are the safety concerns? So I deal with those things first. So somebody with an unstable mental health condition that’s going to be my priority in terms of the time I spend with them. Or if somebody is having angina, then, you know what I mean? The things that are immediately threatening to them their day to day life. *[1A09]* |
|  | Prioritization by presence of symptoms | Usually you have to deal with what’s causing, what is symptomatic at the particular moment and usually diabetes is not. So you may have to kind of work through other issues like arthritis or other real painful conditions or stomach problems or whatever it is that is causing discomfort right away. *[1A07]* |
|  | Prioritization by acuity | And then probably finally, just looking at the history… so If they have had a long history of uncontrolled problems. Whether it be hypertension or whether an uncontrolled A1C for multiple years, it may be easier to tackle something that just come up in the last couple of month, than something that has been long standing. *[1A10]* |
|  | Negotiation | What are your concerns and what do you want to deal with? And here are my concerns. If it’s not a huge list we can accomplish that right? Set up the kind of priorities in terms of the (inaudible 10:49) schedule for coming back. If it’s really complicated and there are a lot of different things happening then it’s going to be a bit of back and forth. We’ll take care of a couple things that we need to take care of today and here’s what I need to take care of. Because it’s just a bit of a compromise. *[1A02]* |
| Goal setting | Conflicting patient and clinician agendas | I routinely see patients who are interesting in gaging around their healthcare which can be frustrating for me, just because I have my idea of what’s important and for whatever reason it may not be their idea of what’s important . *[1A07]* |
|  | Facilitators | |
|  | Understanding patient perspective | So… we worked a little bit on coming up together. Again it's always collaborative. And what she Um... she wanted to do and what the influences were like more on her barriers. [Hmm…]And what she thought would be Um... I guess a small step that maybe… in a way have a… have a good impact on… on her ability for healthy eating. [Right.]So she was finding that she was rushing through her day. And then would just binge at the end of the day and she felt… that she had done before some frequent meals and that really helped her out. So we really just came up with the goal…[Hmm…] …what she wasn’t seeing at the time. That was probably something to really focus on for her. So she went [inaudible 00:10:27] around her… her lunch time and trying to carve out some time in her day that she would take fifteen minutes to take a lunch. [Yeah] I know it sounds really simple but for her that was a big challenge. *[1A05]* |
|  | Interprofessional team approach | Yeah and so if someone comes in and say they don’t want to go on medication but they haven’t done any research, how do you handle that? *[1A09]* |
|  | Patient education | Information and time..**[**Ok] I think are the biggest pieces there. The more informed a patient is, the closer you are in terms of where you are speaking from, and that really does help. *[1A07]* |
|  | Contextualizing patient and provider goals | So I’ll say to them… look you know that’s… that’s great that you want to do this with your lifestyle but I’ll… I’ll still sort of outline and say hey, you know here’s the target… here’s what we’re trying to do with your cholesterol and here’s why. *[1A03]* |
| Approach to decision-making | Educator | Again I would say my approach is still similar to start regardless of their approach. So I view my role as a physician as one providing information and education to the patients and then letting them makes the decision as long as it’s an informed one. So my approach would initially be same… *[1A06]* |
|  | Consultant | This is what’s happening and here it is. I usually have a discussion about you know what some of the different options are? And usually… usually I say to them. Look my job is to sort of give you… I’m like a consultant. I'm going to give you all of the options and you tell me which one you like… you know how you will like to proceed? *[1A03]* |
|  | Decision-maker | Well I would say for... sort of for the general concept of the decisions. You know, I would only be solely involved say in terms of... say we decisive to treat with medication and I... then I wouldn’t expect them to know the options for therapy. **[**Yeah] But In terms of deciding around, like whether they were going to take a medication, deciding whether we were going to initiate insulin, then that would be something we would talk about together. *[1A09]* |
|  | Approach tailored to: | |
|  | Patient preference | I mean, it really varies by patient. I think that some are incredibly proactive and do a lot of research and look into things themselves and want to know everything about it. I think some are much, you know take a passive approach than that. They like hearing what health providers have to say and sort of take that as the first word and most would combine a bit of their own research to attempt to understand what is going on. *[1A07]* |
|  | Patient education level | I think there are 2 different types of patients. The patients who just want you to tell them what to do and they trust your judgment um and then they will try to sort of follow it. And then there are the other patients who have a bit more health literacy, who will do their own research and come up with what they think they should be doing, with obviously, your input... *[1A02]* |
|  | Interprofessional approach | I also work with a dietician. [Hmm…] …Full time. So the two of us… after we see the patient you know… while… we see the patient separately. And then you know… and like I said I will discuss what I think with… needs to be done with the patient… need to change with the patient. And then I’ll also talk to my colleagues… to see if she has any suggestions and pieces that she wants to add in before we send any message to the doctor or we speak to the physician. *[1A01]*  So if a patient wants to start Insulin or hasn’t decided I’ll say… you know. Okay. This is why we need to do this. [Hmm…] If you’re still wavering on it, go home think about it. Talk to your doctor. You know there’s nothing that I do in isolation. *[1A01]* |
|  | Patient engagement | In this day and age I think most of us are fairly patient oriented and guided by patients. You know, I mean, especially with a disease as complex as diabetes. You can’t do it without patient involvement. And especially when you are looking at everything from lifestyle changes to diet and exercise, medications etcetera, etcetera. I mean, these are big changes in people’s lives, so I never found that it works just to tell people what to do. *[1A07]* |
|  | Patient has the final say | As I said these are not children. It’s ultimately their decision. You cannot force anything on them you know. *[1A01]* |
| Shared decision making | Benefits - Active involvement of patient in decision | |
|  | Increase patient accountability | I know that the patients feels there is more ownership on them because they had a choice in making the decision… and then also… so there is accountability because if they go back on that decision we can kind of go back to that conversation and say, well what are you reasons for not doing that at this point. And whether it conflicted with of the reasons why they made the decision to go on it in the first place. *[1A10]* |
|  | Increase adherence | You know, plus this is very long term treatment in many cases. You know, if you are going to start a medication, it’s probably medication that they are going to be on for a long time. They need to be accepting of that and be fully understanding of what they are getting into. *[1A07]* |
|  | Increase patient satisfaction | From what I’ve learned so far about it includes compliance, with the therapy and includes the trust relationship with the physician. When patients feel like that they are more involved in their health care decision it over all improves their overall patient experience with the health care system. I think it it’s an important thing. *[1A02]* |
|  | Challenges: | |
|  | Competing health concerns | I think there are also the issues of competing priorities. This is especially in people who have social pressures, like who live in poverty, inadequate housing, these sorts of pieces that, you know, something like an illness like diabetes it’s not immediately causing symptoms may fall down the priority list because of those other pieces and that sometimes play into it as well. *[1A07]* |
|  | Conflicting agendas | I routinely see patients who are interesting in gaging around their healthcare which can be frustrating for me, just because I have my idea of what’s important and for whatever reason it may not be their idea of what’s important . So you know, there can be a bit of a separation there...and especially when I just feel whatever reason the patient doesn’t understand what’s going on... or doesn’t want to understand what’s going on. *[1A07]* |
|  | - Perception of patient making the wrong decision | It depends on if the goal is one that you know is reasonable and makes sense and what not. So I mean, if it’s a goal that is reasonable, then I would do my best to support that. If it’s a goal that is unreasonable which sometimes happens, then I try to dissuade the patient. If I’ve got somebody that comes in and tells me they are trying to eliminate 20 out of 25 food groups out of their diet because their naturopath tells them they have food sensitivities, I don’t support that goal either and I try to explain evidence and logic. So Again, I do my best, if their goal is reasonable and makes sense, I’ll do my best to support that. *[1A06]* |
|  | Limited time | If I don’t have the time to properly sit back and go through that process with someone… and it’s hard to predict what time you need, right? Sometime just get to the point and it takes 5 minutes. Some people need 45 minutes to go through everything, ask the questions... for me to try to figure out why they are making certain decision or not another one or why they are not getting something. *[1A07]* |
|  | Facilitators | |
|  | Longitudinal relationship | I find a lot of times people are more diligent if they have someone following them through on a regular probably weekly or bi weekly basis, versus the usual 1 to 2 months. *[1A10]* |
|  | Supportive primary care physician | It's tricky. I mean I think that… but… for those patients… but it's trying to remain as non-judgmental as possible. [Hmm…] I always say to myself that at least if the patient comes back… and at least you’re able to sort of keep tabs on them… then if you’re still able to at least measure their blood pressure and you know check on their weight and check in on their overall health… [Yes]…and maintain a relationship with that patient, at least they’re still getting care…[Okay]…and they still have a physician that is caring for their… you know for their health. *[1A03]* |
|  | Considering patient as a whole | I think if you know your patient and you have relationship with them, knowing their context and knowing their… like who they are and where they came from helps you support them in that. *[1A09]* |
|  | Active listening | Um I mean it’s about creating a space where you can listen. Make sure you have heard those goals and that you have set priorities together. *[1A07]* |
|  | Understanding patient perspective | A lot of it is sharing what the personal experience is and the sense of where is the patient coming from with their experience with their diabetes. [Okay] Looking at what their fears and… and what they… Um... are worried about when going on Insulin of making changes because things… like I'm thinking of Insulin but even other meds is that you know weight gain, hypoglycemia, different things like that… that it’s allowing them to voice their concerns so that really understand… so that as a health care professional I understand where they are coming from. *[1A04]* |
|  | Appropriate knowledge base | That sometimes makes the difference. But I think it’s all about education. It’s really important that the patients understands. [Right] Okay? [Okay. Yeah.] If the patient doesn’t understand the disease and what’s going to happen… if they don’t take this seriously, then… you don’t have a baseline to work with. [Hmm…] And I think that’s really important. [Hmm…] You have to have that baseline. They have to know what is happening in their body with the diabetes? What’s causing the problems? What’s going to cause the problems in the future if they don’t start taking care of them? [Hmm…] Then you can move from there. But if you don’t have that knowledge and that understanding they’re basing their decisions on who knows what? *[1A01]* |

**(6)** **Table 5:** **Usability issues**

| **Issue #** | **Issue** | **Change** | **Justification** | **Severity** |
| --- | --- | --- | --- | --- |
| **General** | | | | |
| 1. | Not clear who are health providers | - List the health providers when the term is first introduced. | P1 and P2 were not clear who is considered a health provider. (Comment #1) | High |
| 2. | Not clear how social worker can be of assistance | - Provide some lesser known examples how a role can be of assistance | P1 sought clarification on the role of the social worker (See Comments section) | Moderate |
| 3. | Questions phrased in the negative are difficult to answer | - Rephrase the questions to remove the “double negative” in the answer. See Example #1. | P1 and P2 commented that “double negatives” are made the questions difficult to understand and answer. | High |
| 4. | The web site navigation is confusing. | - Pending heuristic evaluation. | See Comments HP1, HP3. | High |
| **Diet** | | | | |
| 1. | Not clear what constitutes carbohydrates (Q2, Q3). | - Provide some examples. - Pictures that illustrate examples are very effective. | P2 was not clear whether fruits are carbohydrates. Fruits are also listed as high-fibre foods, and it is not clear that they are counted in multiple categories.  P1 suggested including images e.g. of plated meals to simplify the recommendations. | High (If completed by patient alone) |
| 2. | Not clear what constitutes a serving of grains and starches (Q1). | - Provide examples and/or illustrate with images. | Two participants (P1, P3) sought clarification on the question and asked about specific examples (e.g. *P2: “A bagel would be considered how many servings?” P3 commented that it is not clear in which category beans and potatoes belong*). This is a concern as the patients may not be able to complete the questionnaire independently. | High (If completed by patient alone) |
| 3. | Examples of high salt foods not accurate (Q4) | - Find examples of high-salt foods with less ambiguity, or add a qualifier (“most processed foods”) | P3 pointed out that not all canned foods are high salt, such as no-salt-added canned vegetables.  HP3 understood the question to refer to canned and packaged foods only. | High |
| 4. | Inconsistency in the way estimates of frequency are given: “on how many days do you do X” vs “on how many days do you avoid” | - Rephrase the question on salt intake so that it also asks about frequency of intake, similarly to others. | P3 noted this inconsistency and commented that it is harder to answer the questions. P1 and P2 also mentioned the “double negatives” as the things they would change. | Moderate |
| 5. | Not clear what “high” means in reference to salt-intake | - Try to illustrate with examples (e.g. cured and pickled foods) and provide some quantification (e.g. add more than 2 teaspoons of salt to home-made meals) | P3 was unsure how to interpret the question. | Moderate |
| **Medications** | | | | |
| 1. | Is it possible to not have any medications? | - Allow patient to indicate that he/she is not taking any medications | The patient who commented on this was not taking any medications for diabetes even though she was diabetic. | High |
| 2. | Not clear how to proceed if the patient does not have a list of medications ready (e.g. if completing in a waiting room). | - Consider adding “do not know” as an option. | P3 could not remember the names of the medications he was taking. | Low |
| **I Am Most Afraid Of** | | | | |
| 1. | Difficult to select one option | - Consider allowing multiple answers - Consider also some guidance or separating out the more severe (heart attack) from less severe complications (burning pain). | All patients and some health care providers had difficulty with these questions. See comments below.  Also, patients were not able to compare these options. | High |
| **Motivation** | | | | |
| 1. | The question was not well understood. | - Is there a way to eliminate the question and just assume what the underlying motivations are? | Multiple participants were confused by this question. They thought that choosing a fear was similar to expressing a motivation (Don’t want to have a heart attack = don’t want to die). A couple of participants commented simply that the choices were not sufficient “I don’t want to die. And it’s not on there.”  Also, it may be difficult to prepare a list which will cover possible motivations. | High |
| **Goals** | | | | |
| 1. | Navigations in the Goals section is confusing. | - Restructure this section | P1 and HP1 were not sure how to select a goal. A debate ensued. Sue had to intervene. (Comment #1). HP2 could not find the goals at first (the software was subsequently abandoned due to a glitch). | High |
| 2. | The list of goals is inadequate. | - Can the list be expanded? | P3 chose a goal that was available, but really wanted a different goal: “I chose [kidney failure, but because there was nothing like controlling my diabetes, or getting off…” | High |
| 3. | The pages are difficult to navigate. | - Restructure this section | Comments HP1. | High |

Additional usability comments:

- The question asks patients to provide the percent of the time that all medications were taken as prescribed. Note that this question is very difficult to answer well without keeping a log. Most likely, a ballpark answer would be provided, but even so converting it into a percentage may be challenging for patients with lower levels of numeracy. Consider replacing simply with Likert scale options such as: Hardly ever, sometimes, about half of the time, most of the time, almost always.
