# Supplementary material for: Beyond pros and cons – developing a patient decision aid to cultivate dialog to build relationships: insights from a qualitative study and decision aid development
Source: BMC Med Inform Decis Mak. 2019 Sep 18;19:186. doi: 10.1186/s12911-019-0898-5 (PMC6749701; doi:10.1186/s12911-019-0898-5)

**Additional File 2: MyDiabetesPlan – Screenshots**


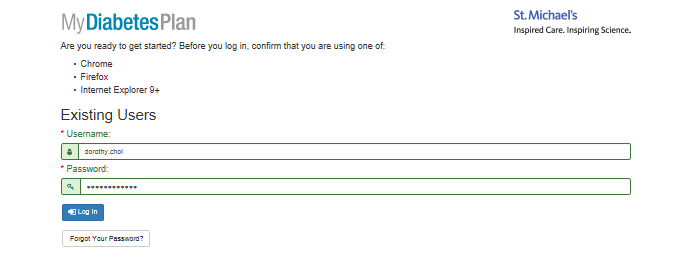


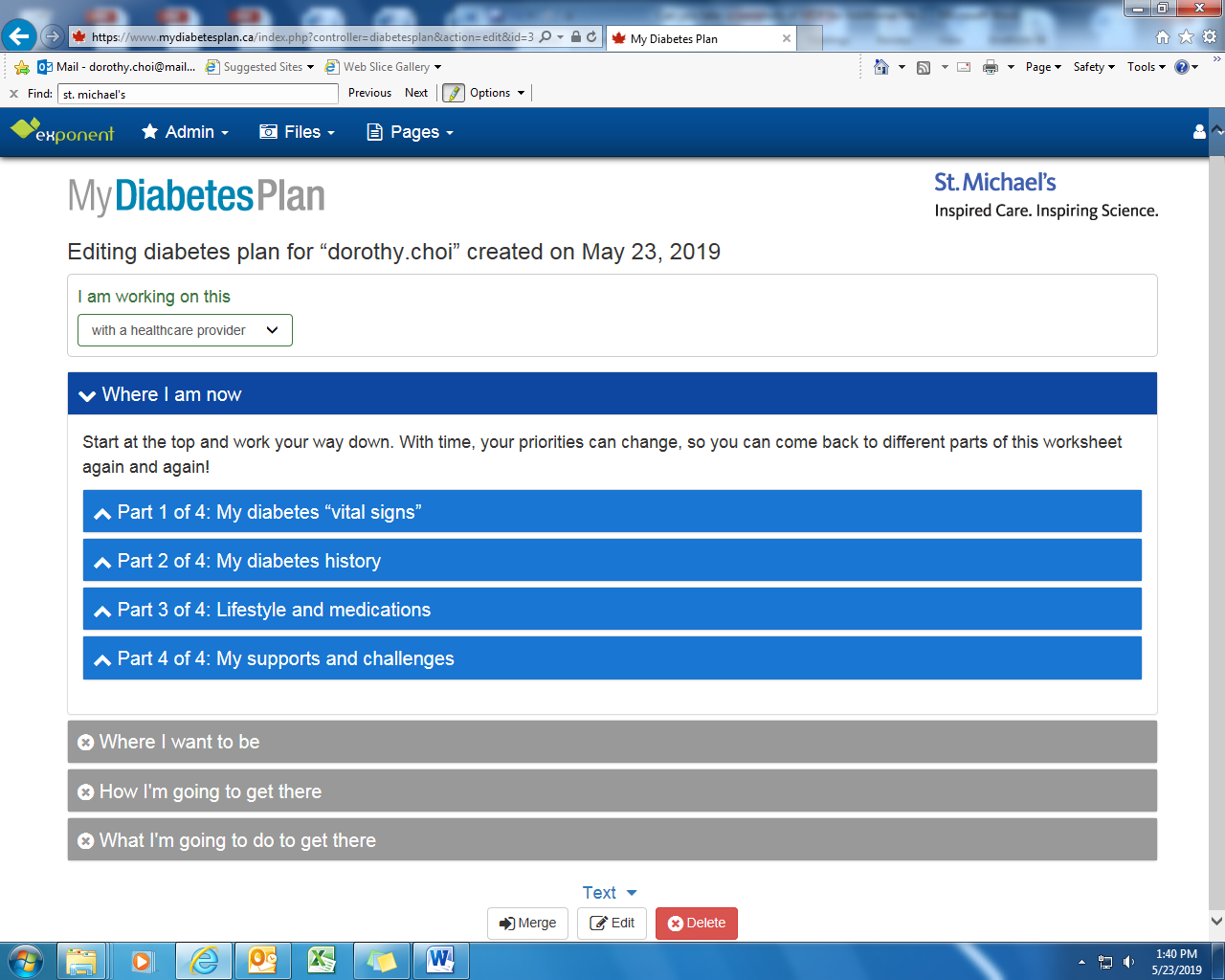


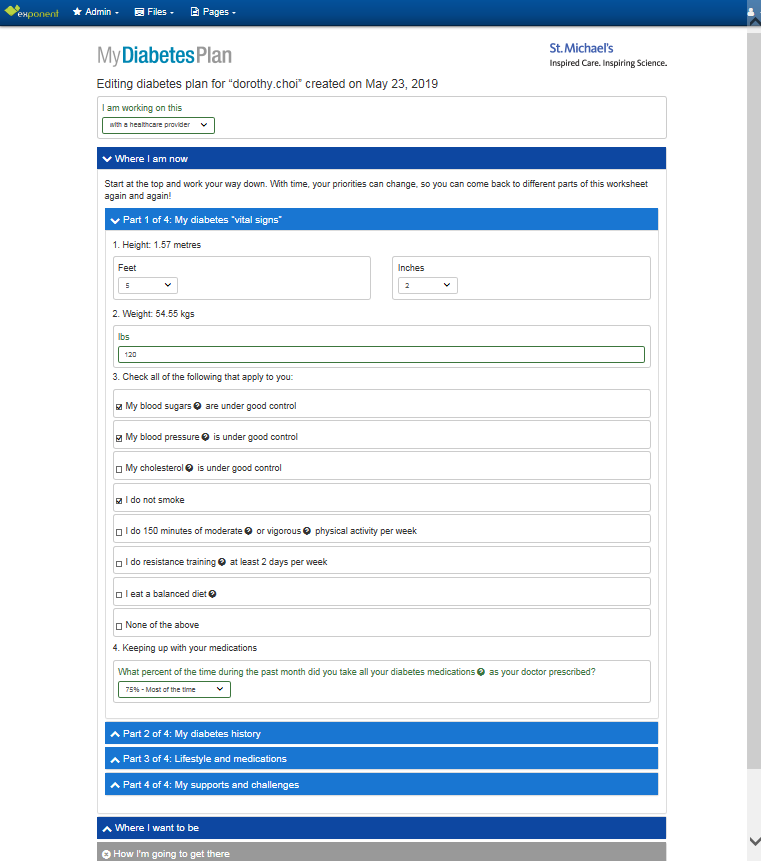


**My Diabetes Plan – Part 1, Section 1: My diabetes “vital signs”**

Patients are inquired on their current height, weight, and baseline diabetes-related health.


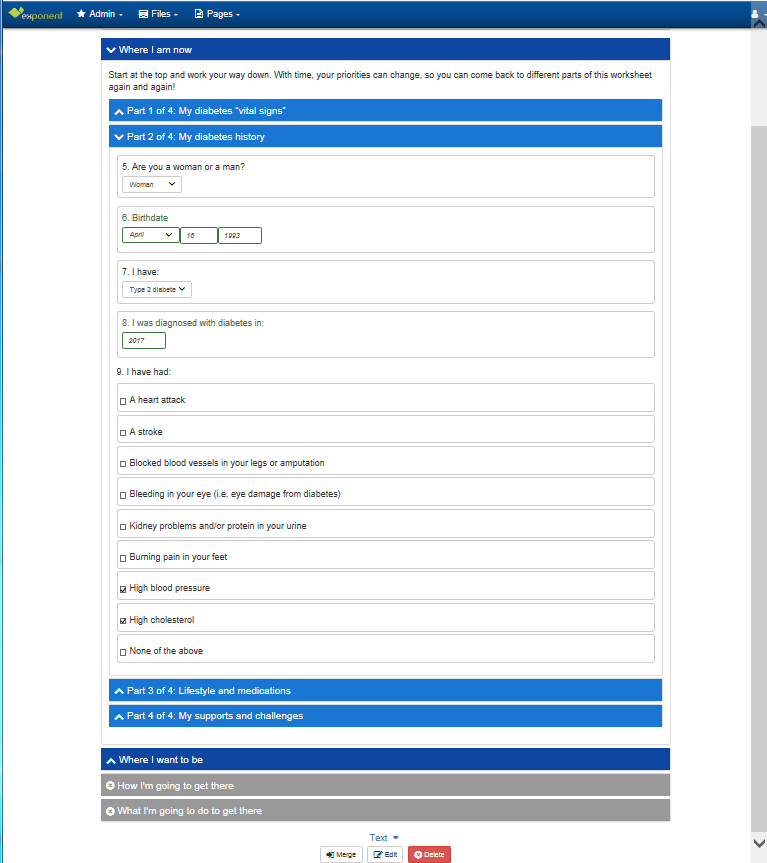


**My Diabetes Plan – Part 1, Section 2: My diabetes history**

Patients are inquired on their gender, diabetes diagnosis and any past diabetes symptoms.


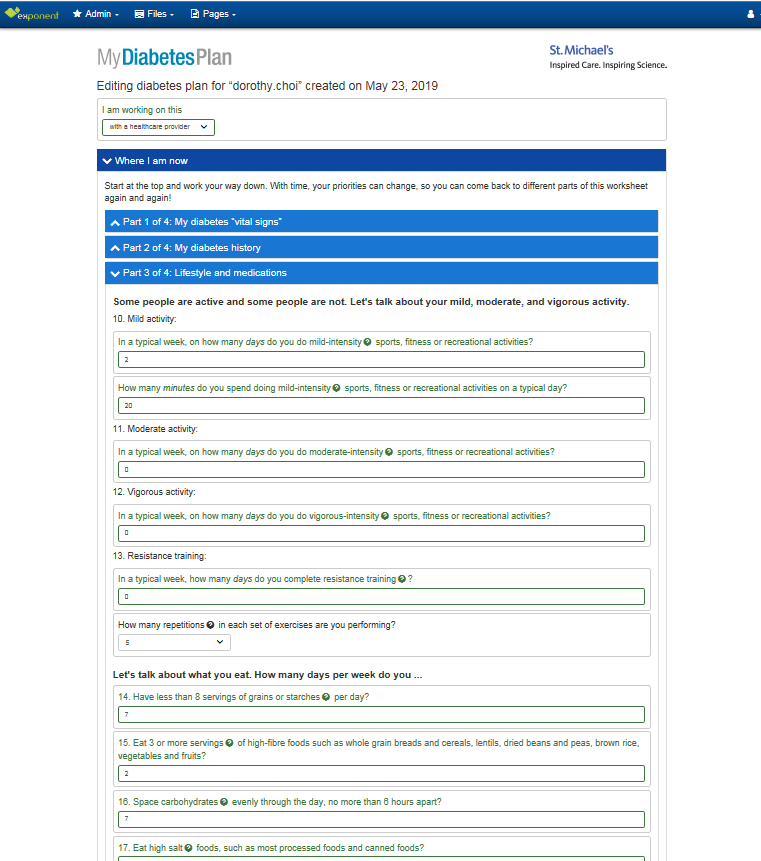


**My Diabetes Plan – Part 1, Section 3: Lifestyle and medications**

Patients are inquired on their physical activity and dietary habits as part of the My Diabetes Plan database.


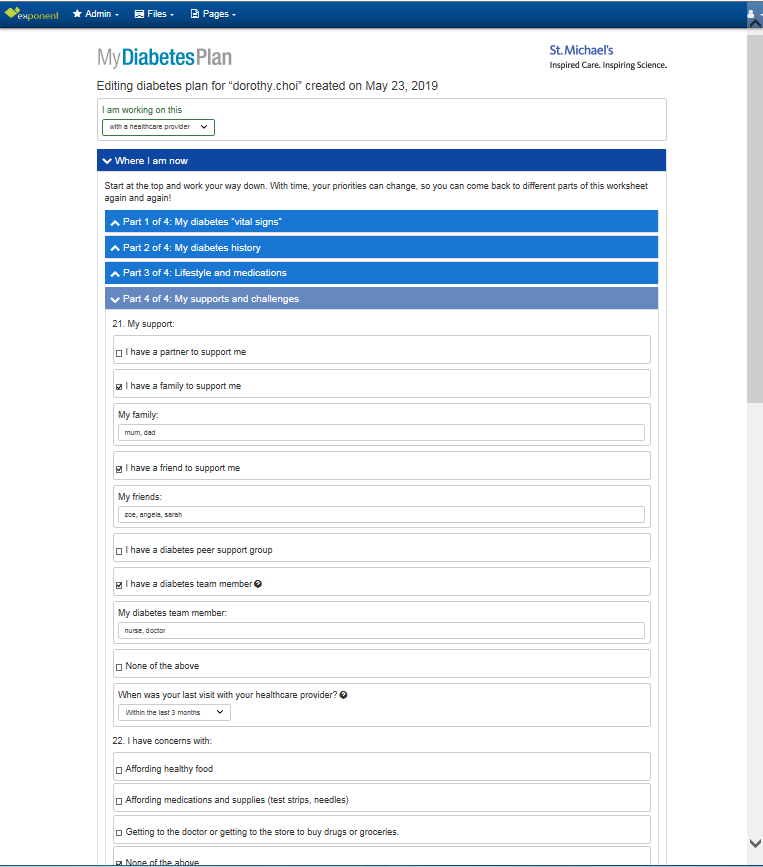


**My Diabetes Plan – Part 1, Section 4: My supports and challenges**

As patients reach this part of My Diabetes Plan, they take a deep dive into their support system, hobbies, and fear complications.


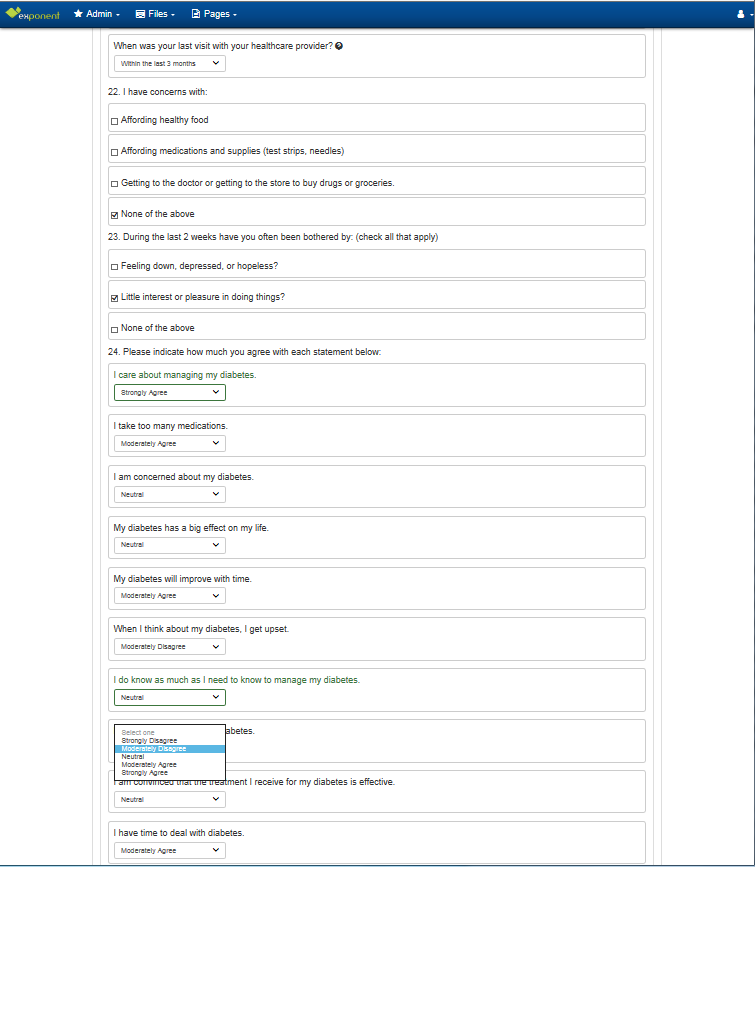

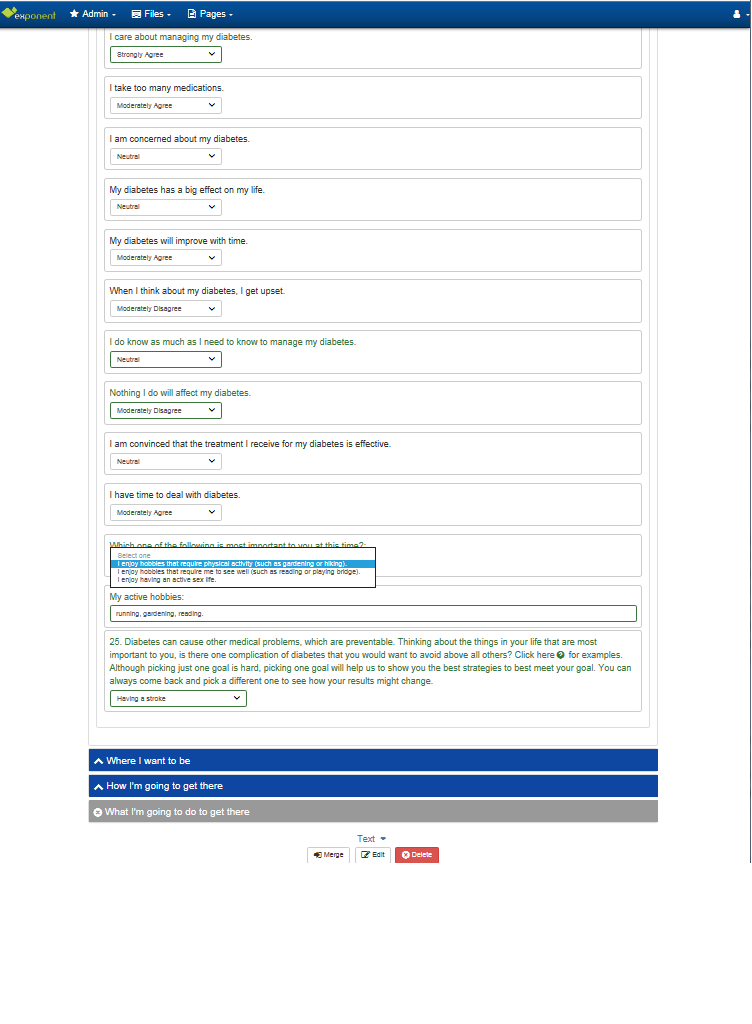

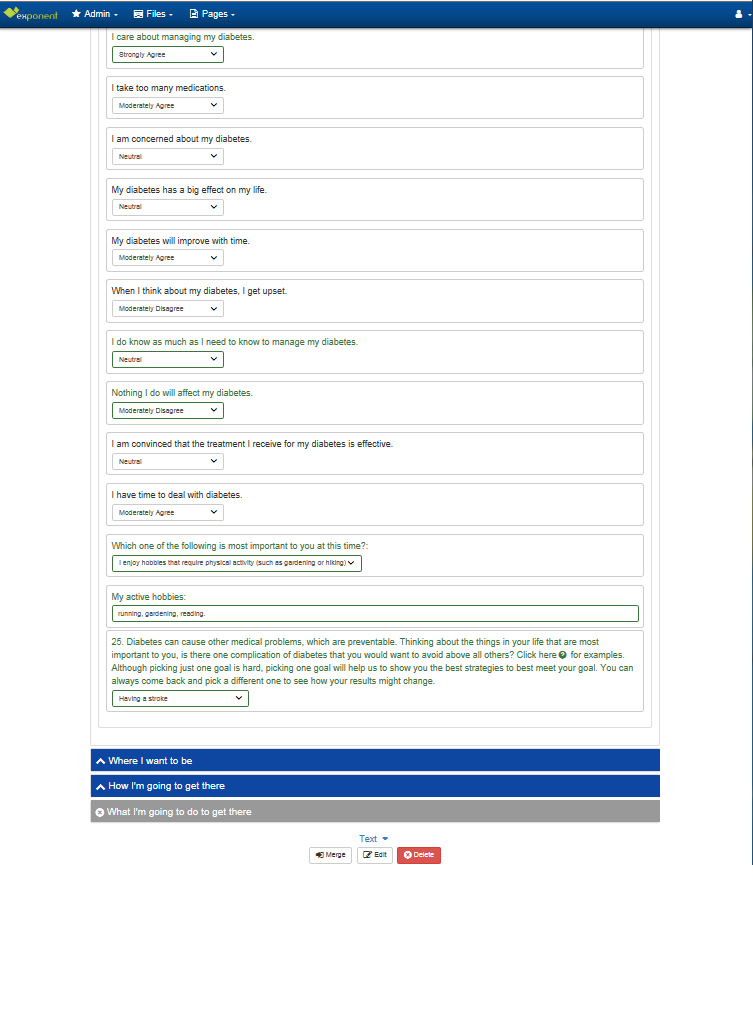


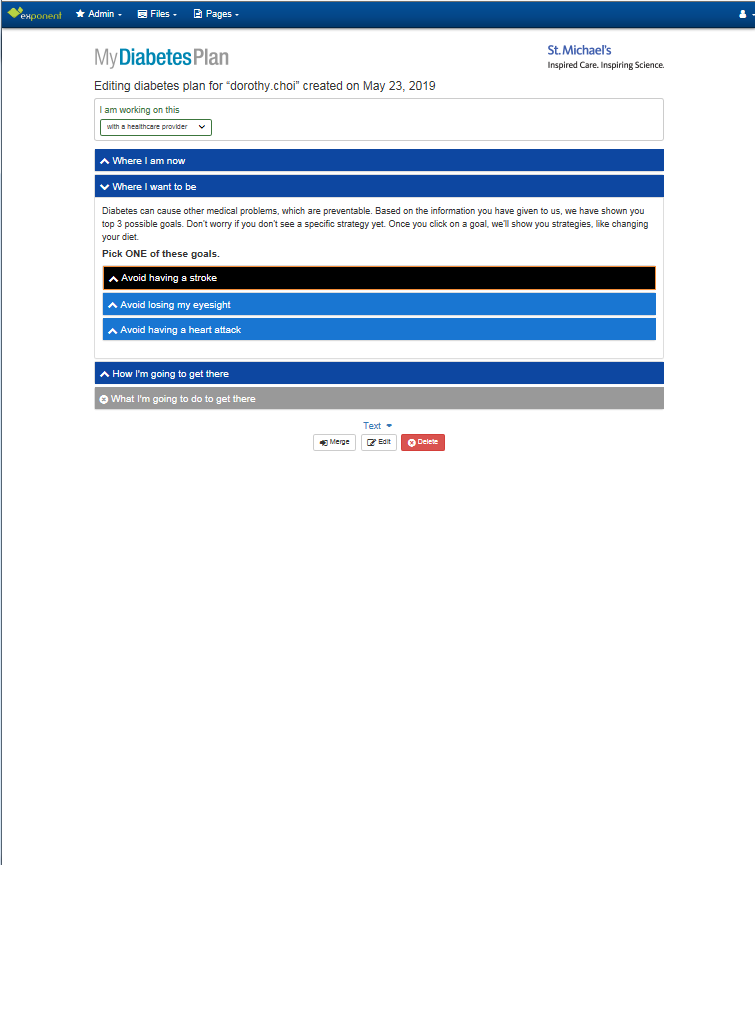


**My Diabetes Plan – Part 2: Where I want to be**

Part 2 of My Diabetes Plan generates top three diabetes-related goals for patients to focus on based on their behavior, preference, and lifestyle responses in part 1.


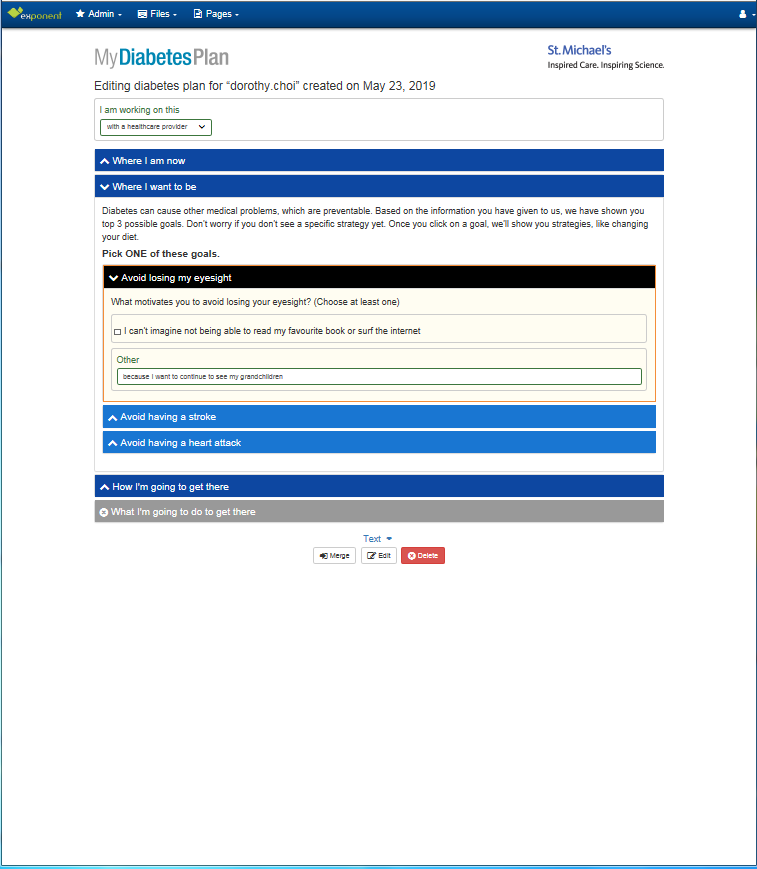


**My Diabetes Plan – Part 2: Where I want to be**

Under each goal, patients can list descriptively what motivates their desire to achieve their diabetes goal – providing context and identity to why they want to improve their overall health.


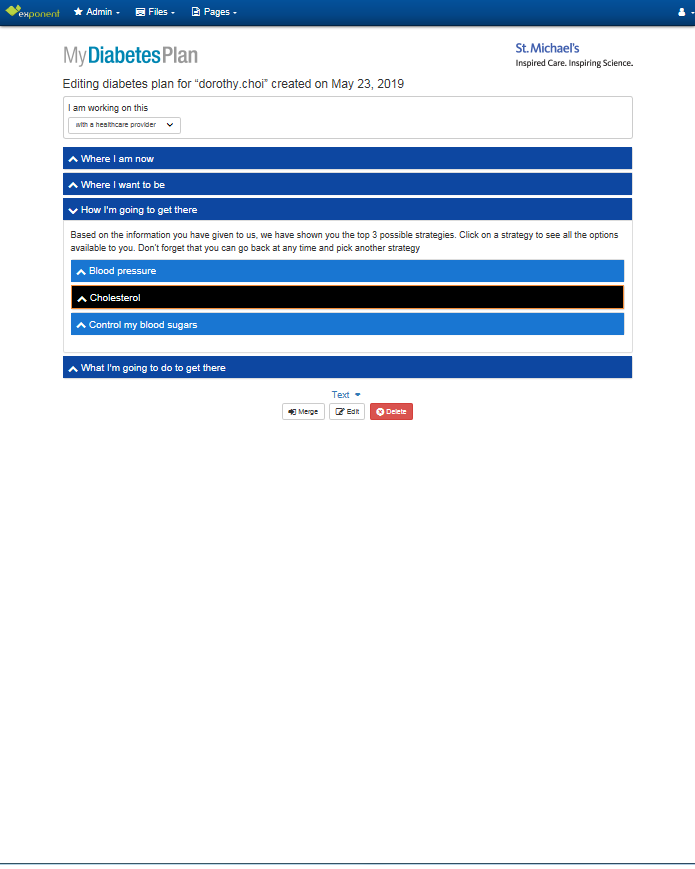


**My Diabetes Plan – Part 3: How I’m going to get there**

Based on patients’ responses in previous sections, part 3 will suggest three best strategies patients can select to fit with their lifestyle, which can help them reach their health goal.


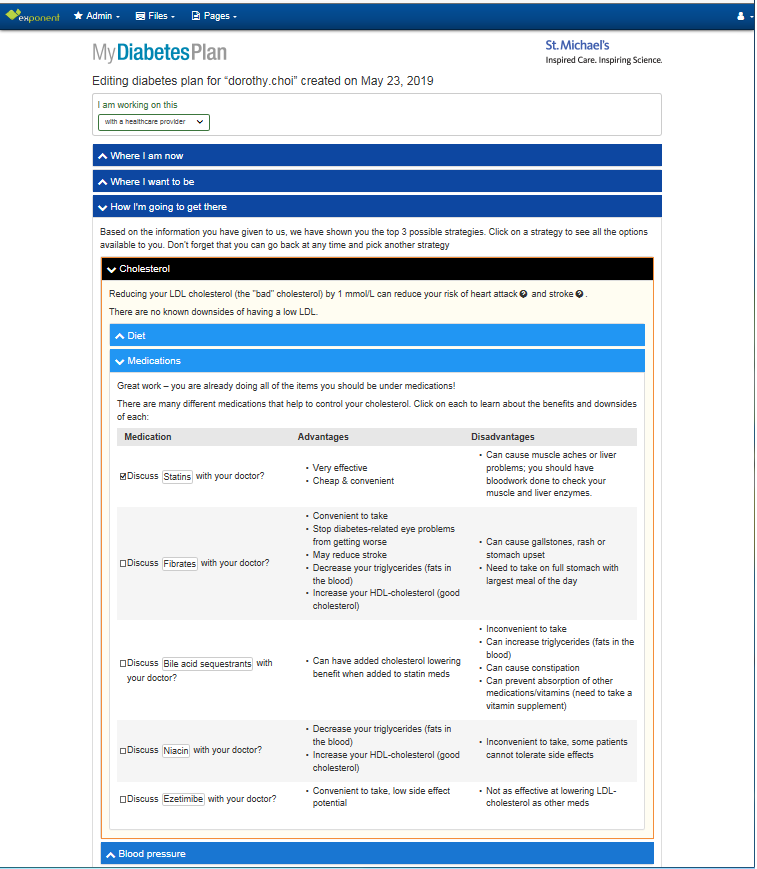


**My Diabetes Plan – Part 3: How I’m going to get there**

From the main strategy selected, patients can select specific pathways available to help them achieve their goal.


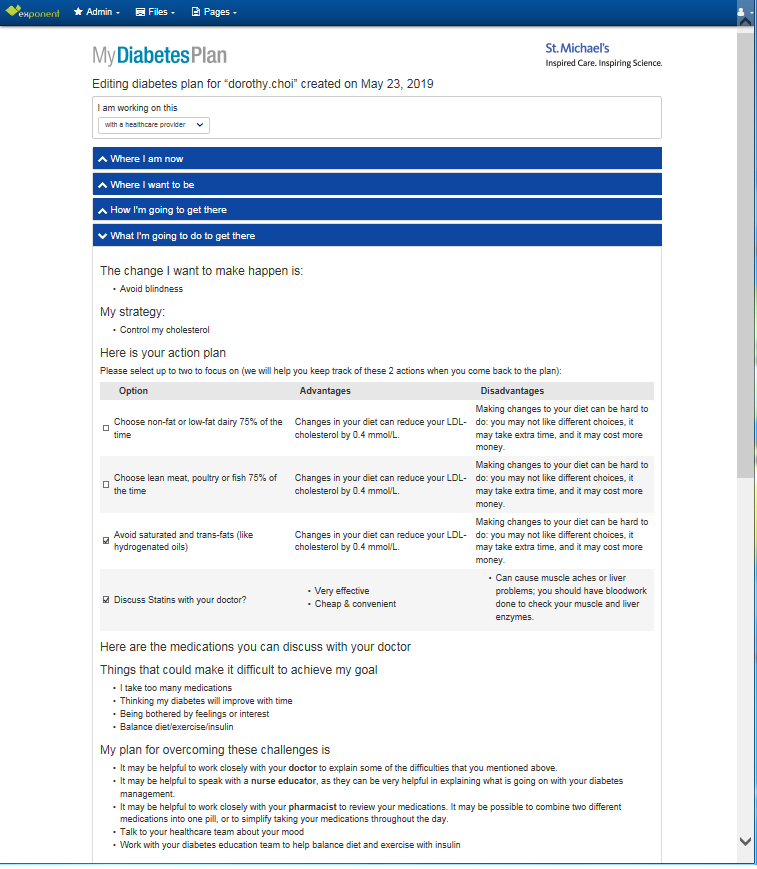


**My Diabetes Plan – Part 4: What I’m going to do to get there**

Once all strategies and goals are selected, an ‘action plan’ will be generated for patients. Additionally, the plan provides an overall summary of patients’ diabetes plan and a follow-up on next appointment date.


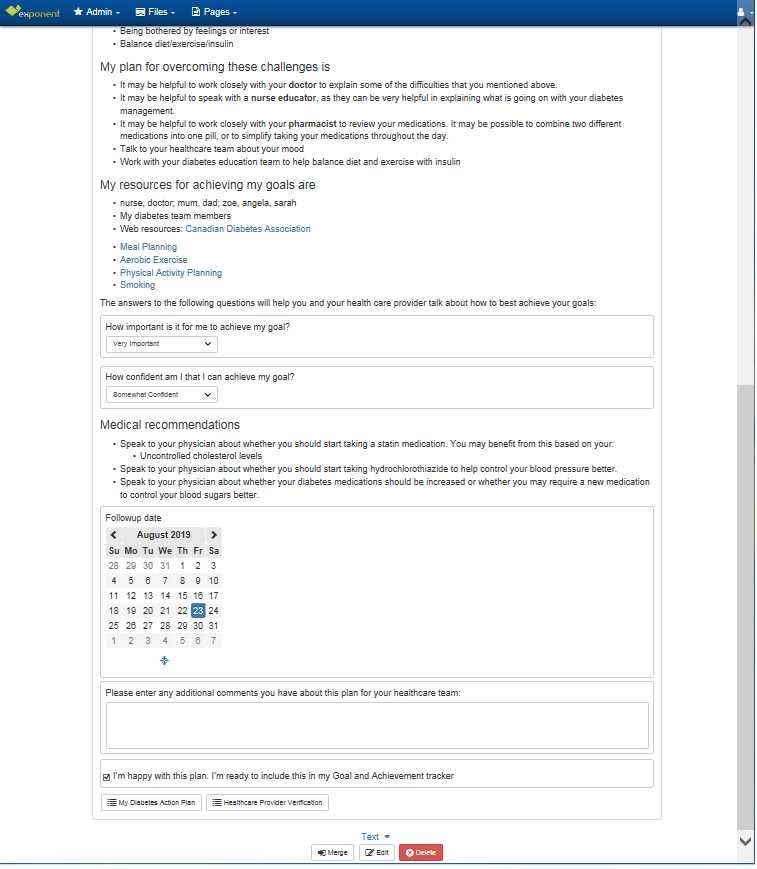

Supplement: Supplementary file 2 — Screenshots of MyDiabetesPlan. (DOCX 803 kb) [file 12911_2019_898_MOESM2_ESM.docx]
